# Whole-genome sequencing of *Aspergillus tubingensis* G131 and overview of its secondary metabolism potential

**DOI:** 10.1186/s12864-018-4574-4

**Published:** 2018-03-15

**Authors:** Elodie Choque, Christophe Klopp, Sophie Valiere, José Raynal, Florence Mathieu

**Affiliations:** 1 Université de Toulouse, Laboratoire de Génie Chimique, UMR 5503 CNRS/INPT/UPS, INP-ENSAT, 1, avenue de l’Agrobiopôle, 31326 Castanet-Tolosan, France; 2 Present address: Unité de Recherche Biologie des Plantes et Innovation (BIOPI-EA 3900), Université de Picardie Jules Verne, 33 rue Saint Leu, 80039 Amiens Cedex, France; 3 Plate-forme Genotoul Bioinfo, UR875 Biométrie et Intelligence Artificielle, Institut National de la Recherche Agronomique, Castanet-Tolosan, France; 4 INRA, US 1426, GeT-PlaGe, Genotoul, Castanet-Tolosan, France

**Keywords:** Black aspergilli, *Aspergillus tubingensis*, Genomics, Secondary metabolism

## Abstract

**Background:**

Black Aspergilli represent one of the most important fungal resources of primary and secondary metabolites for biotechnological industry. Having several black Aspergilli sequenced genomes should allow targeting the production of certain metabolites with bioactive properties.

**Results:**

In this study, we report the draft genome of a black Aspergilli, *A. tubingensis* G131, isolated from a French Mediterranean vineyard. This 35 Mb genome includes 10,994 predicted genes. A genomic-based discovery identifies 80 secondary metabolites biosynthetic gene clusters. Genomic sequences of these clusters were blasted on 3 chosen black Aspergilli genomes: *A. tubingensis* CBS 134.48, *A. niger* CBS 513.88 and *A. kawachii* IFO 4308. This comparison highlights different levels of clusters conservation between the four strains. It also allows identifying seven unique clusters in *A. tubingensis* G131. Moreover, the putative secondary metabolites clusters for asperazine and naphtho-gamma-pyrones production were proposed based on this genomic analysis. Key biosynthetic genes required for the production of 2 mycotoxins, ochratoxin A and fumonisin, are absent from this draft genome. Even if intergenic sequences of these mycotoxins biosynthetic pathways are present, this could not lead to the production of those mycotoxins by *A. tubingensis* G131.

**Conclusions:**

Functional and bioinformatics analyses of *A. tubingensis* G131 genome highlight its potential for metabolites production in particular for TAN-1612, asperazine and naphtho-gamma-pyrones presenting antioxidant, anticancer or antibiotic properties.

**Electronic supplementary material:**

The online version of this article (10.1186/s12864-018-4574-4) contains supplementary material, which is available to authorized users.

## Background

Filamentous fungi of the *Aspergillus* genus, and especially black Aspergilli, have a worldwide distribution and occur on a large variety of substrates. Due to their versatile metabolism, those fungi are one of the most prolific sources of enzymes, organic acids and secondary metabolites (SM) with biomedical and biotechnological interests [1–4]. Already described as non-mycotoxins producer in comparison to *A. niger* [5–9], *A. tubingensis*, which is part of the *A. niger* clade of the black Aspergilli, represents a good alternative for metabolites production in industrial fermentation and is already used for some applications [10–12].

*A. niger* clade is exploited since 1923 for industrial fermentation, production of enzymes / heterologous proteins (α-amylases, cellulase, pectinase) and organic acids (citric acid) used in food, cosmetic and pharmaceutical preparations [1, 13, 14]. Besides, strains of this clade are remarkable producers of secondary metabolites of industrial, agricultural and economic importance. For example, they have the potential to produce compounds such as asperazine, which has antibiotic properties, or Naphtho-Gamma-Pyrones (NGPs), showing antioxidant, anticancer or antibiotic properties [1, 2]. However, they can also produce mycotoxins such as ochratoxin A (OTA) and fumonisin B_2_, which are major concerns in risk assessment in the food chain [5].

It was recently suggested that availability of *Aspergillus* genomic sequences greatly facilitates secondary metabolites (SM) biosynthesis characterization, as expression of most of them is cryptic [15]. Genomic studies allow identification of numerous genes putatively involved in secondary metabolites production. Indeed, filamentous fungi possess a great number of biosynthetic genes such as polyketide synthase (PKS) or non-ribosomal peptide synthase (NRPS). These biosynthetic genes are often clustered with various enzymes-coding-genes (hydroxylase, methyltransferase, cytochrome P450) [1, 16, 17]. However, SM clusters genomic diversity does not reflect the metabolite profiles obtained under laboratory culture conditions, suggesting that a majority of SM biosynthetic pathways are transcriptionally silenced. Those silent biosynthetic pathways could be a rich source of chemically diverse compounds with outstanding potential for industry [16]. Today genomic studies are the best way to get a global view of fungal SM clusters and also offer a good view for their process optimization in industrial production and application.

Sequencing the genomes of a large number of strains from the same species, the same clade or the same genus, enables to run comparative studies on both genomic and biochemical levels [17]. These comparative studies will allow the characterization of biosynthetic pathways based on genes present / absent in organisms regarding their potentiality to produce one specific type of SM. For example, this methodology was applied for OTA biosynthesis genes cluster determination in *A. carbonarius* by sequencing and comparing the genomes of a producing and a non-producing strains [18, 19].

In this study, we report the draft genome of *A. tubingensis* G131 isolated from a French Mediterranean vineyard. This genome has been compared to the recently available *A. tubingensis* CBS 134.48 [20], *A. niger* CBS 513.88 [13] and *A. kawachii* IFO 4308 [21]. Those strains were chosen for their industrial applications. Indeed, *A. niger* CBS 513.88 is an industrial enzyme producer and *A. kawachii* IFO 4308 is a citric acid industrial producer used in koji fermentation for shochu beverage preparation [13, 21]. Those strains cover all types of metabolites that are industrially produced by black Aspergilli. This study focuses on the SM production potential of *A. tubingensis* G131, after a general genome comparison. Results presented here show that the strain does not contain SM clusters required for OTA and fumonisins production, as identified in *A. niger* CBS 513.88 [13, 22]. A complete review of the putative SM gene biosynthetic clusters is also reported. A biochemical analysis shows that *A. tubingensis* G131 produces asperazine and NGPs under laboratory conditions. The comparative genomic analyses conducted on the *A. niger* clade allowed us proposing putative SM clusters involved in asperazine and NGPs production [1, 23, 24].

## Results and discussion

### Genome sequencing

The genome of *A. tubingensis* G131 isolated from a French Mediterranean vineyard [25], with black aspergilli morphological characteristics, was sequenced using Illumina MiSeq technology with a coverage of 143.6X. The genome assembly is approximately 35,18 Mb long and includes 192 scaffolds with an average length of 183,235 bp (Table 1).

**Table 1:** Genome characteristics and predicted features of the assembled *A. tubingensis* strains

Genome	*A. tubingensis* G131 (this study)	*A. tubingensis* CBS 134.48 (de Vries et al. 2017)
Number of scaffolds	192	33
Length of the largest scaffolds	2,380,764 bp	4,803,603 bp
Average length of scaffolds	183,235 bp	1,065,035 bp
Total length of scaffolds	35,18 Mb	35,15 Mb
Sequence Read Coverage	143.6 X	125.7 X
G + C content (%)	50.22%	49.18%
Number of predicted coding genes	10,994	12,322
Predicted proteins average length	524 aa	475aa

According to BUSCO analysis [26], the assembly of *A. tubingensis* G131 genome is robust. Indeed 98,8% of the 4046 groups of genes required for the correct assembly of eurotiomycetes were present in *A. tubingensis* scaffolds assembly (BUSCO results are available in Additional file 1: Table S1).

The genome size of *A. tubingensis* G131 (35,18 Mb) is equivalent to the genome size of *A. tubingensis* CBS 134.48 (35,15 Mb, Table 1) but larger than the *A. niger* CBS 513.88 (34.02 Mb) genome and smaller than the *A. kawachii* IFO 4308 (36,6 Mb) genome [13, 20, 27]. The average shared identity at a nucleic acid level was obtained with ANI calculator [28]. It suggests that the sequenced strain is genetically closer to *A. tubingensis* CBS 134.48 (98.6%) then to *A. kawachii* IFO 4308 (93,75%) and finally to *A. niger* CBS 513.88 (87.62%) but is really far from *A. carbonarius* ITEM 5010 (80.36%) which is also part of the *A. niger* clade. As the genome was closer to *A. tubingensis* CBS 134.48 on a nucleic acid level, a dot plot analysis against this strain was made to order scaffolds assembly (dot plot graphic is available in Additional file 1: Fig. S1). The dot plot alignment does not show any major evolutive event between the two *A. tubingensis* strains.

### Taxonomy

*A. tubingensis* G131 was isolated on a survey on the occurrence of NGPs and OTA producing fungi in grapes [25]. Based on morphological characteristics, this strain was first classified as an *A. niger* strain. Actually, *A. niger* and *A. tubingensis* are the two most common species found in the black Aspergilli. However, due to their close morphological characteristics and often insufficient molecular identification, *A. tubingensis* strain is, most of the time, misidentified as *A. niger* [1].

**Fig. 1:**
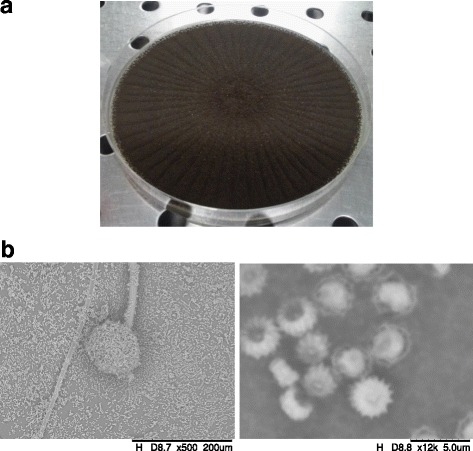
**a** Sporulated mycelium of *A. tubingensis* G131 on CYA plate after 7-day-culture at 28 °C. **b** Scanning electron microscopy of *A. tubingensis* G131 conidia and spores after 7-day-culture at 28 °C (Hitachi TM-3000, Bench SEM)

Colony diameter of *A. tubingensis* G131 is superior at 85 mm after 7 days cultures at 28 °C (Fig. 1a). On these conditions, the filamentous fungus displays high sporulation. Conidial heads are dark brown to black, commonly abundant and slightly floccose as already described in *A. tubingensis* sp. [29]. Conidiophores (70–90 μm) seem to have a limited surface granulation (Fig. 1b). Conidia have a size range of 3–5 μm with a spiny appearance as already described by Samson et al. [29]. However, these morphological characteristics are really close to those of *A. niger*, explaining the confusion previously made by Bouras et al. [25].

**Fig. 2:**
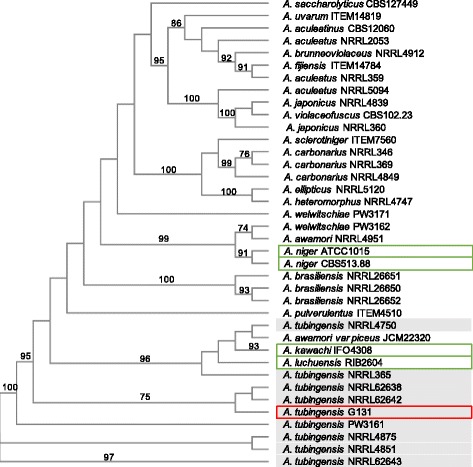
Phylogenetic tree produced from the combined sequence data of three loci (*rpb2*, *benA*, *cam1*) of 38 strains of uniseriate black Aspergillli. Numbers above branches are bootstrap values. Only those above 70% are indicated. The evolutionary history was inferred using the Neighbour-Joining method computed with the Maximum Likelihood Evolutionary method. Grey background highlighted *A. tubingensis* strains; Green frame highlighted the genome used for the comparative analysis of this study; Red frame highlighted the genome sequenced in this study

The phylogenetic analysis performed in this study used several reference genes (*rpb2*, *benA*, *cam1*) and revealed the close relationship between the sequenced strain and other *A. tubingensis* strains [29]. The multilocus analysis was performed on our isolate with 37 reference strains from black Aspergilli (NCBI accession number available in Additional file 1: Table S2). These reference genes were chosen for their percentage of variable and parsimony informative sites per locus as described by Jurjevic et al. [30]. Phylogenetic analysis was conducted first on the three single locus alignments, then the three partial gene sequences were combined in a unique alignment (Fig. 2). The tree with the highest log likelihood is shown. The phylogenic trees of each single locus (Additional file 1: Figures S2, S3, S4) or of the combined loci show almost the same topology. Besides, these results fulfill the requirements of genealogical concordance and phylogenetic species recognition [31], identifying the sequenced strain as an *A. tubingensis*. Besides, phylogenetic results confirm than the sequenced strain is genetically closer to *A. kawachii* IFO 4308 than to *A. niger* CBS 513.88.

### Genome annotation

Genome annotation was performed with Augustus gene prediction software [32]. The annotation predicts 10,994 coding genes, which is less than what is described for the other fungi from the *A. niger* clade: *A. tubingensis* CBS 134.48 (12322), *A. niger* CBS 513.88 (14097) and *A. kawachii* IFO 4308 (11475) [13, 20, 21]. The low number of predicted coding genes for *A. tubingensis* G131 could be linked to the prediction methodology. Indeed, RNA sequencing was also used for genes prediction in the three other genomes. We observe a difference of 1328 predicted genes between *A. tubingensis* CBS 134.48 and *A. tubingensis* G131. Such a discrepancy was already observed between *A. niger* CBS 513.88 and *A. niger* ATCC 1015, which showed a difference of 2882 predictive proteins [14]. Authors affirmed that the difference between the two *A. niger* strains is due to “overprediction in CBS 513.88 / underprediction in ATCC 1015”. To confirm such a hypothesis in the case of *A. tubingensis* strains (*A. tubingensis* CBS 134.48 and *A. tubingensis* G131), we runned the Augustus software (same parameters than for *A. tubingensis* G131) for proteins prediction in *A. tubingensis* CBS 134.48 genome assembly and found 10,652 predicted genes in comparison to 10,994 predicted genes in *A. tubingensis* G131. So, this small difference clearly argued for the overprediction/underprediction hypothesis.

Predicted proteins were annotated through homology search with NCBI BLAST (nr/nt database), Interproscan and Gene Ontology using Blast2GO software [33] (Blast2Go results summary are available in Additional file 1: Figure S5). The average length of predicted proteins is 524 amino acids (Table 1). This value is higher than the average protein length of other black Aspergilli: *A. tubingensis* CBS 134.48 (475aa), *A. niger* CBS 513.88 (442,5 aa) *A. kawachii* IFO 4308 (500,1 aa) [13, 20, 21]. In any case, those results are in agreement with Tiessen et al. who have shown that average protein length in fungi is 487 aa [34].

**Fig. 3:**
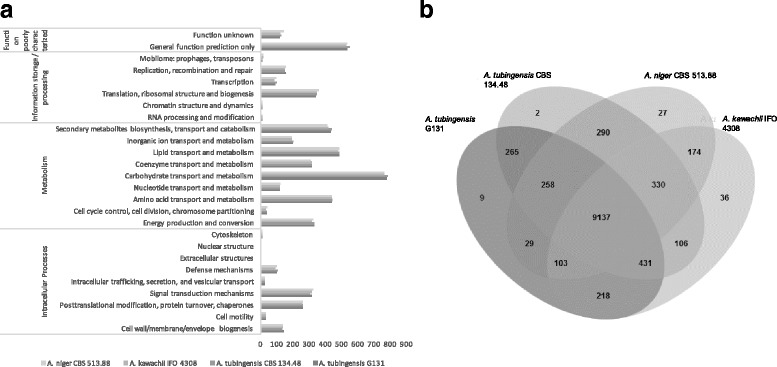
Global comparative genomic analyses of *A. tubingensis* G131, *A. tubingensis* CBS 134.48, *A. niger* CBS 513.88 and *A. kawachii* IFO 4308 **a.** Histogram of KOG distribution of predicted proteins from each genome. **b.** Venn diagram distribution of orthologous gene clusters from the four genomes obtain with OrthoMCL analysis

Eukaryotic orthologous groups [35] (KOG) functional classification of the four compared genomes is shown in Fig. 3a (raw data are available in Additional file 1: Table S3). Four main categories can be distinguished: intracellular processes, metabolism, information storage / processing and function poorly characterized. Results show that function repartition is approximately the same in the four compared genomes. KOG analyses reveal that most of the genes are involved in metabolism: amino acid (≈ 437 proteins / strain), carbohydrates (≈ 771 proteins / strain), lipid (≈ 482 proteins / strain) or secondary metabolites (≈ 422 proteins / strain). In the case of *A. niger* clade, genes involved in metabolism represent 52% of the KOG classification which might support the advantages of this type of strains for industrial application.

OrthoMCL analysis compares amino acid sequences of different genomes through BlastP analyses, clustering them according to their identity (threshold: 50% similarity) [36]. An OrthoMCL analysis was performed on *A. tubingensis* G131, *A. tubingensis* CBS 134.48, *A. niger* CBS 513.88 and *A. kawachii* IFO 4308. Venn diagram of the OrthoMCL results (Fig. 3b) shows a strong homology between the four strains of the *A. niger* clade as 9137 orthologous clusters contain at least one amino acid sequence of each strain. The specific genes, showing no orthology or paralogy relationships between *A. tubingensis* G131, *A. tubingensis* CBS 134.48, *A. niger* CBS 513.88 and *A. kawachii* IFO 4308 were 344, 1331, 1355 and 649 respectively. Moreover, 276 clusters were specific to *A. tubingensis* strains and among them 2 were specific to *A. tubingensis* CBS 134.48 (proteins of unknown function) and 9 were specific to *A. tubingensis* G131. Among them, 6 were of unknown functions, 1 was a putative carboxylesterase, 1 was a DEAD-box RNA helicase and 1 was a putative transposase. Among the 265 orthologous clusters specific to both *A. tubingensis*, 46 show useful functions for SM synthesis: cytochromes P450 (10), dehydrogenases (6), mono/dioxygenases (6), hydrolases (5), aldehyde reductases (5), oxidases (4), carboxylesterases (3), Acetyl-CoA synthases (2), decarboxylase (2), methyltransferases (2), methylesterase (1). Among the 9 orthologous clusters present in *A. tubingensis* G131, no homology with other fungal species genes was found. Moreover, 334 predictive proteins from *A. tubingensis* G131 were not sorted in orthologous clusters and were specific of the studied strain. 23% (78) of these specific proteins seems to have an exogenous origin from different fungal species such as *Penicillium* sp., *Metarhizium* sp., *Aspergillus* sp., *Trichoderma* sp. Interestingly, among those 78 exogenous acquisitions, 23 predictive proteins seem to have been acquired from *Penicillium chrysogenum* and 10 from *Aspergillus oryzae*. As those two genomes (*Penicillium chrysogenum*, *Aspergillus oryzae*) were only assembled in contigs, it is impossible to conclude if they are the result of a single or multiple chromosomal insertion over time.

Considering that many industrial purposes require the production of extracellular enzymes, a secretome analysis was made to estimate the percentage of secreted proteins in each studied genome. Results show that approximately, 10% of each predicted proteome encode secreted proteins for *A. tubingensis* G131 (1107), *A. tubingensis* CBS 134.48 (1391), *A. kawachii* IFO 4308 (1204) and *A. niger* CBS 513.88 (1307). Results show a core secreted proteome composed of 774 protein orthologous clusters between the four strains (at least 1 putative secreted protein from each *Aspergillu*s sp. is present in a cluster - Venn diagram is available in Additional file 1: Figure S6). Regarding biological process, 25,3% of the core secretome acts in secondary metabolic processes, 3,8% acts in cell wall formation and 3,8% acts as response to external stimulus. Besides, 44% of this core secretome shows a SM synthesis function such as hydrolase (28,7%), peptidase (5,9%), oxidoreductase (5,2%) or transferase (4,1%). Finally, 32,5% of the core secretome is required in fungal cell wall or membrane formation and 15,5% is extracellular. Among the extracellular proteins, proteins with SM synthesis functions are mostly found. Regarding secretome specifity of *A. tubingensis* G131, we observe 59 unique secreted proteins. 25% of those proteins seem to have been acquired from various fungal source such as *Penicillum* spp., *Colletotrichum* spp., *Fusarium* spp. or *Neosartorya* spp.. Among those 59 unique secreted proteins, only 3 functions have been found: polysaccharide deacetylase, carboxylesterase and short-chain dehydrogenase reductase. Other putative functions remain unknown as no conserved domain is observable.

### Focus on *Aspergillus tubingensis* G131 secondary metabolism

#### General observation on secondary metabolites biosynthesis

Regarding dot-plot analysis of *A. tubingensis* G131 against *A. tubingensis* CBS 134.48 (Additional file 1: Figure S1), *A. kawachii* IFO 4308 or *A. niger* CBS 513.88 genome assembly, the only genomic re-arrangement observed is a 0.7 Mb inversion observed in *A. niger* CBS 513.88. No duplication, insertion or deletion event were observable in the analysis. It seems that genomic variability between strains and thus their potential to produce specific metabolites were only caused by genomic mutations or genes acquisition / deletion but not chromosomal re-arrangement. This result is in agreement with Lind et al. (2017) which argued that the identity and total number of SM clusters can vary between very closely related species, as for the four studied *Aspergillus* sp., whose genomes exhibit very high sequence and synteny conservation [37].

SM clusters prediction in *A. tubingensis* G131 was done using two available software packages: AntiSMASH and SMURF [38, 39]. AntiSMASH, based on conserved domains search on genome assembly, predicts 81 putative clusters. While SMURF based on fungal clusters comparison from predicted proteins, predicts 66 putative clusters. Inglis et al. [15] already described that both softwares have efficient algorithm for SM clusters prediction. However, there are disparities between predicted cluster boundaries. Results need to be manually refined by taking the farthest one. In *A. tubingensis* G131, 80 SM clusters are predicted after manual refinement. The list of *A. tubingensis* G131 putative SM clusters and their genomic coordinates are available in Additional file 1: Table S4.

The predicted SM clusters of *A. tubingensis* G131 are defined according to their “backbone enzymes” that generate the carbon skeleton of the putative SM. In *A. tubingensis* G131, most of the “backbone enzymes” are polyketide synthase (PKS) or non-ribosomal peptides synthase (NRPS). 31 SM clusters contain sequence coding for a PKS / PKS-like enzymes, 29 SM clusters contain sequence coding for a NRPS / NRPS-like enzymes and 13 SM clusters are hybrid clusters containing sequence coding for both PKS and NRPS enzymes (Table 2). The remaining SM clusters seem to be required in terpene/terpenoid metabolites production as the “backbone enzyme” is a terpene cyclase (9 SM clusters) or a dimethylallyl tryptophan synthase (2 SM clusters). Moreover, we observe that *A. tubingensis* G131 possesses a greater number of PKS / NRPS hybrid SM clusters (13) than the other strains (Table 2).

**Table 2:** Distribution of types of predicted SM clusters, according to their backbone enzymes, predict in *A. tubingensis* G131, *A. tubingensis* CBS 134.48, *A. niger* CBS 513.88 and *A. kawachii* IFO 4308

	*A. tubingensis* G131 (this study)	*A. tubingensis* CBS 134.48 (*de Vries* et al., 2017)	*A. niger* CBS 513.88 (*Andersen* et al., 2011)	*A. kawachii* IFO 4308 (*Yamada* et al., 2016)
DMAT	2	2	2	2
Hybrid	13	7	6	6
NRPS	14	14	14	15
NRPS-like	11	19	15	17
PKS	28	31	29	33
PKS-like	3	6	5	5
TC	9	6	6	6
Total	80	85	77	84

*DMAT* dimethylallyl tryptophan synthase, *Hybrid* PKS / NRPS, *TC* Terpene cyclase

Genomic sequences of *A. tubingensis* G131 SM clusters were individually blasted against *A. tubingensis* CBS 134.48, *A. niger* CBS 513.88 and *A. kawachii* IFO 4308 using NCBI blast on whole genome shotgun (WGS) NCBI data (Table 3). In this study, a SM cluster was considered as highly conserved in another *A. niger* clade strain when BLAST coverage > 80% and identity > 80%. This characterization allows us to discriminate four types of SM clusters: (i) highly conserved in *A. niger*, *A. tubingensis*, *A. kawachii* (36 SM clusters); (ii) highly conserved in *A. tubingensis* and *A. kawachii* (28 SM clusters); (iii) highly conserved in *A. tubingensis* isolates (9 SM clusters); (iv) specific to *A. tubingensis* G131 (7 SM clusters).

**Table 3:** Alignment of SM clusters predicted in *A. tubingensis* G131 with genome assembly of *A. tubingensis* CBS 134.48, *A. niger* CBS 513.88 and *A. kawachii* IFO 4308. cov: coverage; Id: Identity

Group	Cluster	Type	Size (Mb)	Similarity to known clusters						
					*A. tubingensis* CBS 134.48		*A. niger* CBS 513.88		*A. kawachii* IFO 4308	
					Cov (%)	Id (%)	cov (%)	Id (%)	cov (%)	Id (%)
Highly conserved *A. tubingensis A. niger* *A. kawachii*	Cluster 3	NRPS	45,6		99	98	97	87	99	94
	Cluster 6	Terpene	21,3		90	96	93	86	100	92
	Cluster 7	PKS	56,2		97	91	93	89	97	95
	Cluster 9	PKS - Like	12,6		100	99	98	90	100	96
	Cluster 13	PKS	37,7		99	98	84	85	99	92
	Cluster 16	PKS	42,0		100	100	80	85	97	92
	Cluster 17	PKS	51,9		100	99	91	86	99	94
	Cluster 18	NRPS	61,9		98	99	81	91	88	95
	Cluster 23	NRPS	62,7	Aflatrem (cov: 11%)	97	96	82	88	95	93
	Cluster 24	NRPS	41,5		99	97	81	87	93	92
	Cluster 25	NRPS / PKS	77,7		98	98	84	89	96	95
	Cluster 29	NRPS-Like	50,4		99	99	85	92	93	96
	Cluster 32	PKS	35,6		99	99	92	88	97	96
	Cluster 33	PKS	48,0		95	98	80	90	92	94
	Cluster 34	Terpene	22,0		99	96	98	90	99	95
	Cluster 35	NRPS-Like	63,0		99	99	88	91	98	96
	Cluster 36	PKS	50,5		100	99	97	90	99	95
	Cluster 37	Terpene	22,1		99	98	88	89	99	94
	Cluster 38	NRPS-Like	73,4		94	98	92	92	93	94
	Cluster 39	NRPS-Like	54,8		100	99	96	90	99	96
	Cluster 42	NRPS	47,2		100	98	95	89	98	95
	Cluster 43	NRPS	44,0		98	97	85	87	97	95
	Cluster 45	PKS	59,3		99	96	81	91	93	93
	Cluster 49	PKS	68,4		97	95	80	88	95	96
	Cluster 50	PKS	46,5		100	98	92	90	100	95
	Cluster 52	PKS	83,8		99	96	84	85	98	94
	Cluster 53	Terpene	21,6		100	98	96	87	99	95
	Cluster 54	PKS	44,7		99	99	97	90	99	94
	Cluster 56	PKS / NRPS	41,3		98	97	86	85	91	92
	Cluster 62	Terpene	20,8		100	99	88	90	99	96
	Cluster 63	NRPS-Like	56,8		99	99	93	90	98	95
	Cluster 58	NRPS	77,8		100	99	85	83	89	91
	Cluster 67	NRPS-Like	40,9		100	99	91	90	97	95
	Cluster 69	Indole / DMAT	21,4		100	99	84	86	99	93
	Cluster 74	NRPS	75,9		100	99	97	89	99	95
	Cluster 77	NRPS	54,6		99	99	97	91	99	98
Highly conserved *A. tubingensis* *A. kawachii*	Cluster 1	PKS	46,8		100	99	50	91	97	95
	Cluster 2	Indole / DMAT	21,5	Notoamide (cov: 20%)	97	97	77	89	93	94
	Cluster 4	PKS	41,8	Fumonisin (cov: 31%)	100	99	54	81	92	90
	Cluster 5	PKS	42,4		86	98	74	88	92	96
	Cluster 10	Terpene	22,9		88	98	72	88	98	94
	Cluster 11	NRPS-Like	42,7		100	99	74	93	94	92
	Cluster 12	PKS	29,8		100	99	26	82	66	95
	Cluster 15	NRPS-Like	54,5		100	99	79	85	89	93
	Cluster 19	NRPS / PKS	71,4		98	99	43	89	86	97
	Cluster 20	PKS	46,7		98	98	60	86	95	94
	Cluster 22	PKS / NRPS	66,5		100	99	66	88	85	91
	Cluster 26	PKS	40,9		100	99	67	83	81	92
	Cluster 31	PKS	60,1		97	97	75	83	91	92
	Cluster 40	Other	29,9		86	99	65	89	80	93
	Cluster 41	PKS	45,3		96	99	68	91	91	93
	Cluster 47	PKS	57,7		100	99	51	83	83	92
	Cluster 51	NRPS / PKS	102,4	Stigmatellin (cov: 30%)	96	97	76	84	82	93
	Cluster 55	NRPS-Like	53,8		85	96	69	89	87	95
	Cluster 57	PKS	42,1		100	99	53	85	95	92
	Cluster 60	PKS / NRPS	35,1		90	96	77	87	86	92
	Cluster 64	PKS	70,4		99	97	78	83	95	93
	Cluster 66	PKS / NRPS	63,0		100	99	71	88	95	92
	Cluster 68	NRPS-Like	33,8		100	99	n.d.	n.d.	83	91
	Cluster 70	NRPS-Like	52,8		100	99	51	83	84	91
	Cluster 72	NRPS / PKS	43,8		100	99	15	81	85	91
	Cluster 73	NRPS	45,7		99	99	59	88	82	91
	Cluster 75	Terpene / PKS	50,7		100	99	76	92	94	94
	Cluster 76	NRPS	45,5		99	97	75	87	97	92
Highly conserved *A. tubingensis*	Cluster 8	NRPS / PKS	54,0	Isoflavipucine (cov: 12%)	100	99	61	95	25	97
	Cluster 14	NRPS / PKS	66,2		100	99	42	86	60	94
	Cluster 27	NRPS	73,4		100	99	51	90	60	95
	Cluster 30	NRPS	59,6		87	99	58	86	75	93
	Cluster 46	PKS / NRPS	60,7	Shanorellin (cov: 28%)	100	99	53	85	68	92
	Cluster 48	PKS	47,9		97	97	58	82	78	91
	Cluster 59	PKS	46,6		100	99	57	82	70	90
	Cluster 61	NRPS / PKS	79,1		100	99	75	87	69	93
	Cluster 65	NRPS	56,7		83	98	41	84	70	93
Specific to *A. tubingensis* G131	Cluster 21	PKS	82,5	Aflavarin (cov: 40%)	68	95	57	86	77	92
	Cluster 28	PKS-Like	15,3		54	89	48	82	54	92
	Cluster 44	PKS	32,8	TAN-1612 (cov: 80%)	71	96	75	89	72	92
	Cluster 71	Terpene	20,3		76	98	65	86	76	93
	Cluster 78	NRPS-Like	27,5		36	79	39	85	36	78
	Cluster 79	Terpene	22,1		n.d.	n.d.	41	89	93	94
	Cluster 80	PKS	24,1		n.d.	n.d.	30	93	64	88

Among the 7 SM clusters specific to *A. tubingensis* G131, a particular attention should be focused on to cluster 44 (Table 3). According to AntiSMASH database, cluster 44 presents genes similarity of 80% with the biosynthetic cluster of an already known compound: TAN-1612. This similarity suggests that *A. tubingensis* G131 could produce TAN-1612, a neuropeptide Y antagonist, already shown to be produced by *A. niger* CBS 513.88 and *A. niger* ATCC 1015 [40, 41]. Cluster 44 shares genes similarity with all known anthracenone / naphthacenedione compounds produced by fungi: TAN-1612, viridicatumtoxin, asperthecin, neosartoricin and emericellin (Additional file 1: Figure S7). Those compounds show immunosuppressive properties [40]. Those results suggest that *A. tubingensis* G131 could produce anthracenone / naphthacenedione compounds with potential immunosuppressive properties.

The 6 other specific SM clusters present insertion of several kb with no homology in the NCBI nr/nt database. These insertions could confer new functionalities to the SM clusters or could lead to production of putative new secondary metabolites. The schematic representations of these SM clusters are available in Additional file 1: Figure S8. Cluster 21 is composed of 29 putative genes and contains at least 6 transporters and 2 transcription factors. The PKS enzyme is poorly conserved (BlastP maximum identity: 62%) and is composed of 4 known PKS domains: KS – AT – PT (DH) – PP. The Product Template (PT) domain of the PKS as a dehydratase (DH) function suggests Claisen cyclization of the natural product through loss of hydroxyl radicals. The presence of a gene coding a putative acyl-CoA dehydrogenase in the cluster suggests that one precursor of the putative natural compound could be acetyl-CoA. Moreover, the PP-binding domain of the PKS and a gene coding for a HMG-CoA reductase might suggest that a fatty acid chain could be attached and modified during the biosynthesis of the natural compound. Despite of this biosynthetic information, the structure of the putative compound cannot be estimated due to too many hypothetical proteins in the cluster.

#### Focus on putative mycotoxins production

The production of OTA and fumonisins by black Aspergilli has already been demonstrated, especially in *A. niger* [1, 22, 25]. OTA, for example, is produced in variable amounts depending on black Aspergilli species. *A. carbonarius* is known to consistently produce large amounts of OTA whereas only 6–10% of the *A. niger* produce it [1]. Black Aspergilli can also produce fumonisin B2 and fumonisin B4 [22, 42]. Based on the current knowledge about the biosynthesis of these mycotoxins, BLAST analyses were performed to check the mycotoxins production potentiality in *A. tubingensis* G131 strain, a methodology that was already described for different genomic works on *Aspergillus* sp. [27, 43, 44].

##### Genomics features of putative OTA biosynthesis in *A. tubingensis* G131

Through biochemical analyses, Bouras et al. [25] showed that *A. tubingensis* G131 does not produce OTA under certain conditions. According to literature, the main black Aspergilli OTA producer is *A. carbonarius*, which is also from the *A. niger* clade. Recently, the genome of *A. carbonarius* Acv3, an atoxigenic strain, was sequenced. Comparative genomic analyses with OTA producer *A. carbonarius* ITEM 5010 were performed focusing on genes known to be involved in OTA biosynthesis [18]. Cabañes et al. suggested that the atoxigenicity of *A. carbonarius* Acv3 could be linked to the high mutation rate observed in a specific PKS of this strain, affecting its function. It was shown that biosynthesis of OTA in *A. niger* CBS 513.88 is mediated by a PKS (An15g07920) which is clusterized with genes coding for a cytochrome P450 and an NRPS [13]. This cluster identified in *A. niger* CBS 513.88 genome is missing in *A. niger* ATCC 1015, a citric acid producer without OTA biosynthesis [14]. In addition, a 21-kb region of the *A. niger* CBS 513.88 OTA cluster is also absent in *A. luchuensis* NBRC 4314 and in *A. kawachii* IFO 4308 genomes, two strains which do not produce OTA [21, 27].

In this study, all the genetic information (genes and clusters sequences) available on OTA biosynthesis was blasted against *A. tubingensis* G131 genome (NCBI accession number and blast results are available in Additional file 1: Table S5). For example, *A. tubingensis* G131 does not contain an orthologue of An15g07920. However, although the backbone enzyme is missing, two genes from the putative OTA clusters in *A. niger* and *A. welwitschiae* have orthologues in *A. tubingensis* G131. These genes are coding for hypothetical proteins with none described key functions in OTA production. Moreover, based on their position in the genome, they are not included in one of the predictive SM cluster, suggesting that there is no backbone enzyme close to these genes. Genomic analyses suggest that *A. tubingensis* G131 could not produce OTA. This is in agreement with what was already published about the OTA non-productivity of *A. tubingensis* strains [1, 45].

In addition, one of the predicted proteins of *A. tubingensis* G131 shows strong identity (NCBI TBlastN – Cov: 100%; Id: 97.3%) with an ochratoxinase from *A. niger* WK143 (NCBI accession: KJ854920.1), suggesting that the strain could be a putative biocontrol agent to limit OTA concentration in food chains.

##### Genomics features of putative fumonisin biosynthesis in *A. tubingensis* G131

In contrast with OTA production, both *A. niger* CBS 513.88 and ATCC 1015 genomes include putative homologues of *Fusarium verticillioides* fumonisin genes and their production of Fumonisin B2 has been confirmed [1, 22, 42]. The fumonisin gene cluster of *A. niger* contains at least 14 *fum* genes [43]. A previous study showed that *A. luchuensis* NBRC 4314 genome only contains orthologues for *fum1* and *fum15* with high identity: 68 and 72%, respectively [27]. Moreover, others FUM were predicted to be encoded by *A. luchuensis*, but the proteins only shared 20–43% identities and the orthologue genes of such proteins were distributed throughout the *A. luchuensis* genome. Susca et al. [43] suggested that fumonisin production was widespread among black Aspergilli. They demonstrated that nonrandom partial deletion of fumonisin cluster has occurred multiple times in several black Aspergilli genomes. Considering *A. tubingensis* G131, the predictive SM cluster 4 shows partial homology with the *A. niger* fumonisins cluster. This cluster was identified by NCBI BlastN and it matches on approximately 13 kb among the 54 kb of the fumonisin cluster (Additional file 1: Figure S9). This represents only intergenic region (query coverage: 31%; identity: 81%, blast results are available in Additional file 1: Table S5). Except *fum1* and *fum15*, none of the *fum* genes found in *A. niger* has an orthologue in *A. tubingensis* G131 (Additional file 1: Table S5). These results suggest that *A. tubingensis* G131 could not produce fumonisins.

#### Focus on SM production with potential for industrial applications

A LC-MS analysis was performed on a methanol extract obtained from 7-days culture of *A. tubingensis* G131 grown on CYA plate at 28 °C (Table 4). According to these data and those from Nielsen et al. [1] on biochemical compounds produced by *A. niger* clade, *A. tubingensis* G131 has the capacity to produce asperazine and 7 NGPs: fonsecin (TMC-256B1), rubrofusarin, aurasperones B, C, D, E and F. Therefore, a genomic search was carried out to identify the putative SM clusters involved in asperazine and NGPs biosynthesis.

**Table 4:** LC-MS analysis of a methanol extract obtained from 7-days culture of *A. tubingensis* G131 on CYA plate at 28 °C. RT: Retention Time; MM: Monoisotopic Mass

LC-MS analysis (*this study*)				secondary metabolites of *A. niger* clade (*Nielsen* et al. *2009*)		Elementary composition	Secondary Metabolite
RT (min)	[M-H]^+^	[M-H]^−^	UV/Vis	MM (Da)	UV/Vis		
17	291	289	230; 276; 333; 405	290.08		C_15_H_14_O_6_	Fonsecin (TMC-256B1)
25	665	663	225; 275; 300	664.28	225; 275; 300	C_40_H_36_N_6_O_4_	Asperazine
27.5	272	271	223; 282; 333; 405	272.07	225; 278; 328; 415	C_15_H_12_O_5_	Rubrofusarin
31	593	592	234; 282; 334; 405	592.16	236; 283.5; 323; 334; 412	C_31_H_28_O_12_	Aurasperone C
33	575	573	228; 281; 321; 334	574.15	213; 281; 320; 334	C_31_H_26_O_11_	Aurasperone F
35	607	605	232; 282; 330; 405	606.16	235; 282; 321; 334; 410	C_32_H_30_O_12_	Aurasperone B
37	589	587	224; 280; 407	588.13	213; 281; 315; 332; 403	C_32_H_28_O_11_	Aurasperone E
	557	555		556.14	235–240; 280; 320–325; 380	C_31_H_24_O_10_	Aurasperone D

##### Putative SM cluster for asperazine biosynthesis

**Fig. 4:**
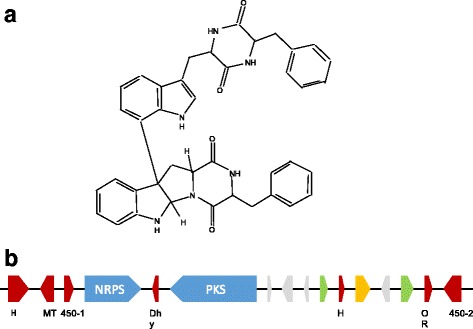
Asperazine biosynthesis in *A. tubingensis* G131 – **a.** Asperazine structure **b.** Schematic representation of the putative asperazine SM cluster (cluster 46). Arrows indicate putative genes in the cluster and its direction indicates forward and reverse strand. Blue: gene coding for a backbone enzyme, Red: gene coding for an enzyme with known function, Green: gene coding for a transcription factor, Yellow: gene coding for a transporter, Grey: gene coding for an hypothetical protein. Below each biosynthetic gene, the putative function is indicated. Dhy: dehydratase, H: hydrolase, OR: oxidoreductase, MT: methyltransferase, 450: cytochrome P450

Asperazine, a diketopiperazine of the ditryptophenaline family (Fig. 4a), is a complex dimer alkaloid. It was first isolated from a marine-derived *A. tubingensis*, initially reported as *A. niger* by Varogulu et al. [23]. According to Nielsen et al. [1], this compound is a valuable chemical marker allowing to distinguish *A. tubingensis* from *A. niger*. Currently, asperazine can be synthetically produced [46, 47]. The elucidation of synthetic steps gives crucial indication/information about required enzymatic functions for its in vivo production and makes it easier to trace genes encoding biosynthetic enzymes. Moreover, some biosynthetic steps for production of other diketopiperazines are already known. For example, the biosynthetic gene cluster for ditryptophenaline production in *A. flavus* is described [48]. It is composed of three genes coding for a NRPS (*dtpA*), a methyltransferase (*dptB*) and a cytochrome P450 (*dptC*). It was also demonstrated that production of fumitremorgin in *A. fumigatus* required 3 cytochromes P450 (*ftmE*, *ftmC* and *ftmG*) for cyclization, hydroxylation of the indole ring and hydroxylation of fumitremorgin C, respectively [49]. Among the 80 SM clusters predicted in the *A. tubingensis* G131 genome, only one contains genes coding for a NRPS, a methyltransferase and a cytochrome P450: cluster 46 which we consider potentially involved in asperazine production. According to the SM clusters classification established here, cluster 46 belongs to the “highly conserved in *A. tubingensis* strains” category (Table 3). Indeed, this cluster is poorly conserved in *A. niger* CBS 513.88 (cov: 53%; id: 85%) and *A. kawachii* IFO 4308 (cov: 68%; id: 92%). This is in agreement with the conclusion of Nielsen et al. proposing asperazine as a marker for *A. tubingensis* strains classification [1].

As shown Fig. 4b, SM cluster 46 is composed with genes coding for 2 transcription factors, 1 transporter, 2 backbone enzymes (1 PKS and 1 NRPS), a methyltransferase and 2 cytochromes P450. It also contains genes coding for enzymatic functions such as hydrolase, dehydratase or oxidoreductase activities. The conserved domains of the putative NRPS, PKS, methyltransferase and the 2 P450 cytochromes are available in Additional file 1: Figure S10.

Blast analyses of Cluster 46 were performed with all data available on diketopiperazines biosynthesis. This cluster shows homology with the fumitremorgin biosynthesis cluster identified in *A. fumigatus* Af293 (cov: 44%; id: 89%, Additional file 1: Table S5). Moreover, NRPS and methyltransferase of Cluster 46 show homology with DptA and DptB of *A. flavus* respectively (Additional file 1: Table S5). However, none of the two cytochromes P450 present in the SM cluster 46 shows homology with DptC. Indeed, asperazine dimerization is based on a C3-C7 linkage which is not observed in such compounds so far [50]. Kishimoto et al. proposed that biosynthesis of dimeric diketopiperazines, such as asperazine, is catalyzed by P450 cytochromes with relaxed substrate specificity [50]. This suggest that cytochromes P450 putatively required for asperazine production could be different from the one of ditryptophenaline biosynthesis in *A. flavus* and that cluster 46 could be the SM cluster for asperazine biosynthesis in *A. tubingensis* G131. However, to confirm the involvement of cluster 46 in asperazine synthesis, genetic experiments should be performed.

##### Putative SM cluster for NGPs biosynthesis

**Fig. 5:**
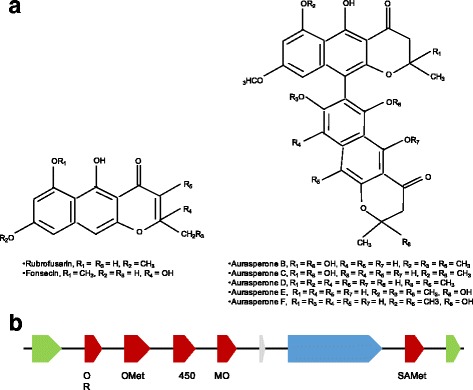
NGPs biosynthesis in *A. tubingensis* G131 - **a.** Schematic representation of the putative NGP SM Cluster (Cluster 16). The representation was manually designed with information obtained from cluster prediction (AntiSMASH, SMURF), genes prediction (Augustus) and Annotation (AntiSMASH) **b.** Schematic representation of the putative NGP SM cluster (cluster 16). Arrows indicate putative genes in the cluster and its direction indicates forward and reverse strand. Blue: gene coding for a backbone enzyme, Red: gene coding for an enzyme with known function, Green: gene coding for a transcription factor, Grey: gene coding for an hypothetical protein. Below each biosynthetic gene, the putative function is indicated. SAMet: S-adenosylmethionine-dependent methyltransferase; OMet: O-methyltransferase; MO: monooxygenase; OR: oxidoreductase; 450: cytochrome P450

As already described, fungi from the *A. niger* clade are NGPs producers [1]. Depending of the species, produced NGP dimers can vary: aurasperones, asperspyrones, nigerasperones or fonsecinones [2]. LC-MS analysis of a methanol extract indicates that *A. tubingensis* G131 is able to produce the NGPs monomers fonsecin and rubrofusarin and NGPs dimers members of aurasperones (Fig. 5a). These secondary metabolites are synthesized through cyclization of acetyl-CoA and malonyl-CoA precursors by a PKS identified as AlbA / PksP on *A. niger* ATCC 11414 and *A. niger* N402 [51, 52]. This PKS known as the “yellow conidial pigmentation PKS” is now well characterized in diverse fungi, especially in *Aspergillus* spp.. It is described that this PKS is involved in the formation of YWA1, which is a common precursor of both NGPs and DHN-melanin pigments [52]. After this step, little is known about NGPs biosynthesis [2].

To identify the SM cluster that could be involved in NGPs biosynthesis in *A. tubingensis* G131, a BlastP analysis was performed to find the putative *A. tubingensis* G131 AlbA / PksP. Two candidates were selected: PKS of SM cluster 54 and PKS of SM cluster 16 (Distance tree results of the two predicted PKS putatively involved in NGPs synthesis are available in Additional file 1: Figure S11). According to the SM clusters classification established here, both clusters belong to the “highly conserved in *A. tubingensis, A. niger, A. kawachii* strains” category (Table 3), which corresponds to the assumption that all strains from the *A. niger* clade are able to produce NGPs.

Distance trees show that the predicted PKS from cluster 54 has a strongest homology to AlbA / PksP (id: 96%) than the predicted PKS from cluster 16 (id: 87%). Moreover, genomic sequence of cluster 54 shows homology with the genomic region of *A. tubingensis* CBS 134.48 coding for SM cluster Asptu1.62 (JGI information). This putative SM cluster corresponds to a unique PKS, which is non-clusterized in *A. tubingensis* CBS 134.48. Comparing AntiSMASH and SMURF prediction for cluster 54 in *A. tubingensis* G131, a mismatch can be observed. SMURF identifies this PKS as a non-clusterized PKS, validating the result previously obtained on *A. tubingensis* CBS 134.48 [20]. Those results are in agreement with results previously obtained in different *A. niger* strains showing that the PKS involved in biosynthesis of both NGPs and DHN-melanin is non-clusterized [2, 51, 52]. For this reason, we suggest that cluster 54 PKS is homologue to AlbA / PksP and most likely the entire SM cluster is not solely involved in NGPs biosynthesis.

PKS from cluster 54 and 16 are orthologues and share the same conserved domains organization: SAT-KS-AT-ACP-ACP-TE (Additional file 1: Figures S11 & S12). The same conserved domains organization was observed for two PKS in *A. niger* ATCC 1015: AlbA and PKS44 [16]. According to this result, we suggest that cluster 16 PKS could lead to the same type of cyclization than AlbA / PksP and be involved in the synthesis of the same type of intermediate. Looking further into functions of cluster 16 predicted proteins (Fig. 5b), it appears that genes of this cluster encode for 2 transcriptional factors, 1 Cytochrome P450, 1 monooxygenase, 2 methyltransferases, 1 oxidoreductase and 1 protein of unknown function. According to the structure of NGPs produced by *A. tubingensis* G131, after PKS cyclization, the main enzyme activities required for their synthesis are hydroxylation, o-methylation and dimerization. Hydroxylation is catalyzed by monooxygenases, o-methylation by o-methyltransferases and some cytochromes P450 or other types of oxidoreductases could catalyze dimerization. Cluster 16 possesses all the required functions for the biosynthesis of NGPs. This is not the case of cluster 54, which lacks oxidoreductases. Moreover, among the two transcription factors identified in cluster 16, one is a zinc finger transcriptional factor which could be involved in the regulation of the entire cluster, and the other is a NmrA-type transcriptional factor, specific to oxidoreductase negative regulation which could be involved in dimerization control. For these reasons, we hypothesize that cluster 16 could be the putative SM cluster for the biosynthesis of NGPs in *A. tubingensis* G131. However, this hypothesis needs to be validated by genetic analyses.

The variability of equivalent SM clusters through the *A. niger* clade (Genetic distance and genes similarity of *Aspergillus* sp. with cluster16 are available in Additional file 1: Figure S13) could be explained by the different NGP types produced by the strains, and conserved domains required for their synthesis. However, a deeper comparative genomic and biochemical analysis should be performed to link the differences between cluster sequences and the putative NGPs produced.

## Conclusions

An increasing amount of knowledge about black Aspergilli is becoming available, largely derived from genome sequencing projects. In this study, we report the genome draft of *A. tubingensis* G131 and highlight its secondary metabolism potential. Indeed, 80 SM biosynthetic gene clusters were identified in the genome assembly. Comparisons of these SM clusters with genome assemblies of *A. tubingensis* CBS 134.48, *A. niger* CBS 513.88 and *A. kawachii* IFO 4308 allow identification of seven clusters described for the first time and putatively unique. However, sequencing of other *A. tubingensis* strains, future comparative genomic analyses and biochemical and genetic characterization will be necessary to validate and confirm the singularity of these putative SM clusters.

Genes coding enzymes involved in OTA and fumonisins synthesis in black Aspergilli were systematically searched. This genomic analysis allows to confirm biochemical results previously obtained by Bouras et al. [25] and showing that *A. tubingensis* G131 is not able to produce these two types of mycotoxins. Besides, the genomic analyses performed here suggest that *A. tubingensis* G131, isolated from a French Mediterranean vineyard, is a good candidate to produce natural compounds with interesting biological properties such as asperazine (antibiotic) and NGPs (antioxidant, anticancer, antibiotic). Genomic studies also suggest that the strain can produce anthracenone / naphthacenedione with putative immunosuppressive properties [40, 46].

## Methods

### Culture and genomic DNA extraction

*A. tubingensis* G131 was isolated from a French Mediterranean vineyard, classified into *Aspergillus* section *Nigri* (morphological characterization) and described as non-ochratoxigenic (biochemical characterization) [25]. The strain was cultured 3 days at 28 °C under shaking condition 120 rpm on PDB medium. The fungal mycelial mat was harvested and ground into a fine powder with liquid nitrogen and conserved at − 80 °C until used. Genomic DNA was extracted from this powder with a protocol adapted from [53]. 150 mg of mycelial powder was transferred to a pre-cooled Eppendorf tube with 700 μl of CTAB solution (1% CTAB; 100 mM NaCl; 100 mM EDTA pH 8; 20 mM Tris-Cl pH 8), homogenized by vortex and incubated 1 h on ice. Genomic DNA was then extracted using two successive Phenol/Chloroform/Isoamyl Alcohol (Sigma) washes, precipitated with anhydrous ethanol and suspended in nuclease free water. Genomic DNA concentration and quality were estimated using Nanodrop 2000 (Eppendorf).

### Genome sequencing and assembly

The Illumina MiSeq platform was used for the whole-genome shotgun sequencing of *A. tubingensis* G131. Pair-ends sequences of 2 * 300 bp were produced. The raw sequence data were trimmed with trim_galore v0.4.0 [54] and a quality control was performed with fastqc [55]. Genome assembly was performed with discovardenovo-52,488 [56]. Statistics of this assembly were collected using the assemblathon_stats.pl script [57].

### Genome annotation and taxonomy

#### Genome annotation

Genome annotation was done using Augustus gene prediction software [32] using *A. nidulans* as a reference. Then, predicted proteins were annotated by homology search using TblastN on NCBI and BlastX on Interproscan databases through Blast2GO software (default parameters) [33]. Blast2GO results allow comparing annotation in the Gene Onthology databases to identify putative molecular functions and cellular processes acquired by the strain.

#### Taxonomy

Taxonomy was performed through molecular identification by alignment of reference genes RNA Polymerase Subunit 2 (RPB2), beta-tubulin (BenA) and Calmodulin (Cam1) as described in [29, 58, 59]. DNA sequences were BlastN on NCBI database and then aligned using Clustal omega algorithm [60]. Each locus was aligned separately and then concatenated in a super-gene alignment, as described in [29] used to generate the phylogenetic tree, with clustal phylogeny [60] based on Neighbor-Joining method. Bootstrap values were computed from 100 replications of the bootstrap procedure using phylogeny.fr and added to the phylogenetic tree [61]. All positions containing gaps and missing data were eliminated from the dataset (complete deletion option).

### Genome comparative analyses

#### Comparative wide comparison of orthologous clusters

Genome wide analysis of orthologous clusters was done through the interactive plateform OrthoVenn [36] using *A. tubingensis* G131, *A. tubingensis* CBS 134.48 [20], *A. niger* CBS 513.88 [13] and *A. kawachii* IFO 4308 [21] predicted proteomes.

#### R2Cat

Syntheny analyses between *A. tubingensis* G131 and *A. tubingensis* CBS 134.48 were addressed with r2cat software [62].

#### KOG analyses

Clusters of Orthologous Groups of proteins (COGs) from the sequenced genomes of *A. tubingensis* G131, *A. tubingensis* CBS 134.48 [20], *A. niger* CBS 513.88 [13] and *A. kawachii* IFO 4308 [21] were analysed using the COG database [35].

#### Secretome analysis

The predictive secretome was obtained using SignalP with default parameters [63]. Each genome analysis was addressed independently.

#### Identification of secondary metabolites biosynthetic gene clusters

The identification of SM biosynthetic gene clusters was performed both with SMURF software [38] and AntiSMASH [39]. SMURF is based on cluster search on the annotated proteome and found putative clusters through conserved domain homology. On the contrary AntiSMASH works directly on the genome scaffolds assembly and find biosynthetic gene clusters through homology with known clusters. The clusters obtained with both methodologies were compared through blast analyses and concatenated for the final SM biosynthetic gene cluster prediction. Concatenation is based on the choice of SM cluster boundaries, to have the longest clusters.

### Culture and secondary metabolites profiling

#### Culture

*A. tubingensis* G131 was cultured at 28 °C, on CYA plates (CYA – 30 g/L Saccharose, 15 g/L agar, 5 g/L Yeast Extract, 2 g/L NaNO_3_, 0.25 g/L KCl, 0.25 g/L MgSO_4_.7H_2_O, 0.005 g/L FeSO_4_.7H_2_O, 0.5 g/L K_2_HPO_4_, 0.001 g/L ZnSO_4_.7H_2_O and 0.0005 g/L CuSO_4_.7H_2_O). After 7 days, the agar plate containing mycelium and spores was covered with methanol. The mycelium / methanol mix was incubated 20 min at room temperature and sonicated at 50 Hz for additional 20 min. After sonication, the mycelium / methanol mix was filtered once on 113 V grade Whatman filter paper. The obtained filtered extract was conserved at 4 °C in the dark until HPLC analysis.

#### LC-MS analysis

HPLC were run on Ultimate 3000 DIONEX device, equipped of a Prontosil 120–5-C18 column (150 × 4.6 mm, SH: 50 μm). Samples were conditioned in 1 ml untainted glass vials, filtered with PTFE 0.45 μm filter. 10 μL was injected for analysis. Acquisition was done between 200 and 600 nm while column was maintained at 30 °C. Compounds separation was obtained by a 40 min linear gradient from acetonitrile-water-acetic acid (30: 69.9: 0.1, *v*/v) to pure acetonitrile, followed by a 5 min isocratic step of pure acetonitrile and then equilibrated during 5 min to acetonitrile-water-acetic acid (30, 69.9: 0.1, v/v), at a flow rate of 0.8 mL/min.

Mass spectrometry analyses were realized on a MS/MS *Q-TRAP* system (*Applied Biosystems*®) with a ESI as ionization source. Analyses were runned in both positive and negative modes.

## Additional file

Additional file 1: **Table S1.** BUSCO analysis of *A. tubingensis* G131 scaffolds assembly. **Figure S1.** Dot Plot analysis between *A. tubingensis* G131 and *A. tubingensis* CBS 148.33 to order the scaffold assembly. **Table S2.** Accession number (NCBI) of sequences used for phylogenetic analysis. **Figure S2.** Phylogenetic tree produced from *Rpb2* partial gene sequence of 38 strains of black aspergilli. **Figure S3.** Phylogenetic tree produced from *Cam1* partial gene sequence of 38 strains of black aspergilli. **Figure S4.** Phylogenetic tree produced from *BenA* partial gene sequence of 38 strains of black aspergilli. **Figure S5.** Blast2GO statistics summary. **Table S3.** KOG analysis of *A. tubingensis* G131, *A. tubingensis* CBS 134.48, *A. kawachii* IFO 4308 and *A. niger* CBS 513.88. **Figure S6.** Venn Diagram of secretome analysis obtained through SignalP and OrthoMCL analysis. **Table S4.** SM Clusters coordinates of the scaffolds assembly. **Figure S7.** Genes similarity between SM cluster 44 from *A. tubingensis* G131 and others known fungal biosynthetic cluster. (Results obtained with AntiSMASH software). **Figure S8.** AntiSMASH schematic representation of the conserved domains in SM clusters unique in *A. tubingensis* G131 and their similarities with other SM clusters identified in various fungi (AntiSMASH results). **Table S5.** Blast results for Ochratoxin A, Fumonisins and asperazine biosynthesis in *A. tubingensis*. **Figure S9.** Schematic representation of Cluster 4 presenting small homology with fumonisin cluster. **Figure S10.** Repartition of conserved domains in predictive enzymes coding genes of the asperazine cluster obtained with CD search. **Figure S11.** Conserved domain (CD-Search) and Distance tree results obtained from NCBI BlastP analysis of predicted PKS of *A. tubingensis* G131 putatively involved in NGPs synthesis. **Figure S12.** Clustal Omega Alignment of predicted PKS from SM Clusters 54 and 16 of *A. tubingensis* G131. **Figure S13.** Genes similarity between Cluster 16 from *A. tubingensis* G131 with other known fungal genomes assembly. (PDF 2680 kb)

## Abbreviations

COGs: Clusters of Orthologous Groups of proteins
Cov: coverage
CYA: Czapech Yeast Agar
Id: identity
NGPs: Naphtho-Gamma-Pyrones
NRPS: Non-ribosomal peptide synthase
OTA: Ochratoxin A
PKS: Polyketide Synthase
SM: Secondary Metabolite
TC: Terpene cyclase
WGS: Whole Genome Shotgun
