# Supplementary material for: Whole-genome sequencing of Aspergillus tubingensis G131 and overview of its secondary metabolism potential
Source: BMC Genomics. 2018 Mar 15;19:200. doi: 10.1186/s12864-018-4574-4 (PMC6389250; doi:10.1186/s12864-018-4574-4)

SUPPLEMANTARY DATA

Whole-genome sequencing of *Aspergillus tubingensis* G131 and overview of its secondary metabolism potential

CHOQUE Elodie[a,b], KLOPP Christophe[c], VALIERE Sophie[d], RAYNAL José[a] and MATHIEU Florence[a*]

[a] Université de Toulouse, Laboratoire de Génie Chimique, UMR 5503 CNRS/INPT/UPS, INP-ENSAT, 1, avenue de l'Agrobiopôle, 31326 Castanet-Tolosan, France

[b] Present address: Unité de Recherche Biologie des Plantes et Innovation (BIOPI-EA 3900), Université de Picardie Jules Verne, 33 rue Saint Leu, 80039 Amiens Cedex, France

[c] Plate-forme Genotoul Bioinfo, UR875 Biométrie et Intelligence Artificielle, Institut National de la Recherche Agronomique, Castanet-Tolosan, France

[d] INRA, US 1426, GeT-PlaGe, Genotoul, Castanet-Tolosan, France

* Corresponding author: florence.mathieu@ensat.fr ; Phone : +33534323935; Fax : +33534323901

Supplementary Table 1. BUSCO analysis of *A. tubingensis* G131 scaffolds assembly

BUSCO Categories	Numbers of groups	Percentage
Complete BUSCOs (C)	**3999**	**98.8%**
Complete & single BUSCOs (S)	**3989**	**98.6%**
Complete & duplicated BUSCOs (D)	**10**	**0.2%**
Fragmented BUSCOs (F)	**39**	**1%**
Missing BUSCOs (M)	**8**	**0.2%**
Total BUSCOs groups searched	**4046**	

Supplementary Figure 1. Dot Plot analysis between *A. tubingensis* G131 and *A. tubingensis* CBS 148.33 to order the scaffold assembly.

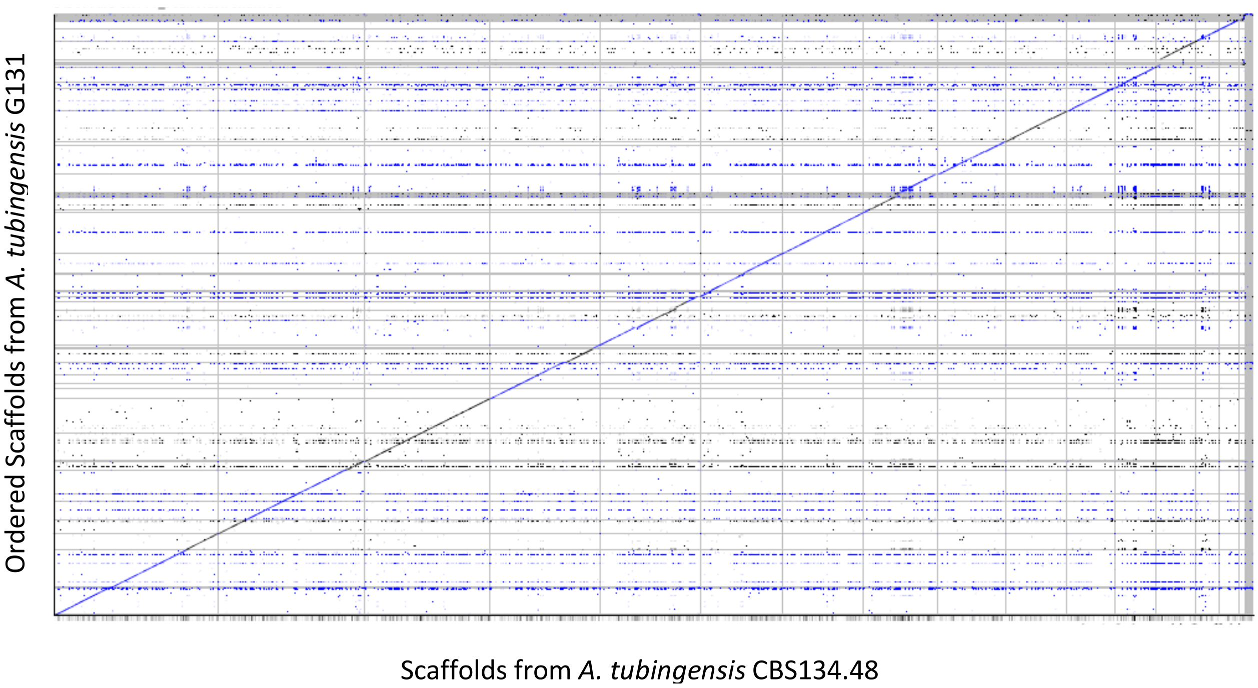

Supplementary Table2. Accession number (NCBI) of the sequences used for phylogenetic analysis

Strain		RPB2	Cam1	BenA
Aspergillus brunneoviolaceus	NRRL 4912	EF661045	EF661147	EF661105
Aspergillus carbonarius	NRRL 4849	EF661069	EF661168	EF661100
	NRRL 369	EF661068	EF661167	EF661099
	NRRL 346	EF661067	EF661166	EF661098
Aspergillus ellipticus	NRRL 5120	EF661051	EF661170	EF661122
Aspergillus fijiensis	ITEM 14784	HE984374	HE984426	HE984411
Aspergillus heteromorphus	NRRM 4747	EF661050	EF661169	EF661103
Aspergillus japonicus	NRRL 360	EF661047	EF661141	EF661082
	NRRL 4839	EF661049	EF661142	EF661081
Aspergillus pulverulentus	ITEM4510	HE984368	HE984423	HE984408
Aspergillus saccharolyticus	CBS 127449	HF559235	HM853554	HM853553
Aspergillus sclerotioniger	CBS 115572	HE984369	FN594557	FJ629304
Aspergillus uvarum	ITEM 14819	HE984380	HE984435	HE984421
Aspergillus violaceofuscus	CBS 10223	HF559234	FJ491697	FJ491686
Aspergillus welwitschiae	PW3171	LC000582	LC000569	LC000556
	PW3162	LC000574	LC000561	LC000548
Aspergillus awamori	NRRL 4951	KC796423	KC796371	KC796358
Aspergillus aculeatinus	CBS 121060	HF559233	EU159241	EU159220
Aspergillus aculeatus	NRRL 5094	EF661046	EF661148	EF661083
	NRRL 359	EF661043	EF661146	EF661106
	NRRL 2053	EF661044	EF661145	EF661107
Aspergillus brasiliensis	NRRL 26650	EF661062	EF661159	EF661079
	NRRL 26651	EF661064	EF661160	EF661094
	NRRL 26652	EF661063	EF661161	EF661095
Aspergillus tubingensis	NRRL 4750	EF661052	EF661152	EF661087
	NRRL 4875	EF661055	EF661151	EF661086
	NRRL 4851	EF661054	EF661150	EF661085
	NRRL 365	EF661053	EF661149	EF661084
	PW3161	LC000573	LC000560	LC000547
	NRRL 62638	KC796431	KC796380	KC796364
	NRRL 62642	KC796435	KC796384	KC796368
	NRRL 62643	KC796436	KC796385	KC796369

Supplementary Figure 2. Phylogenetic tree produced from *Rpb2* partial gene sequence of 38 strains of black aspergilli. The evolutionary history was inferred using the Neighbour-Joining method computed with the Maximum Likelihood Evolutionary method. Grey background highlights *A. tubingensis* strains; Green frame highlights the genome used for the comparative analysis of this study; Red frame highlights the genome sequenced in this study.

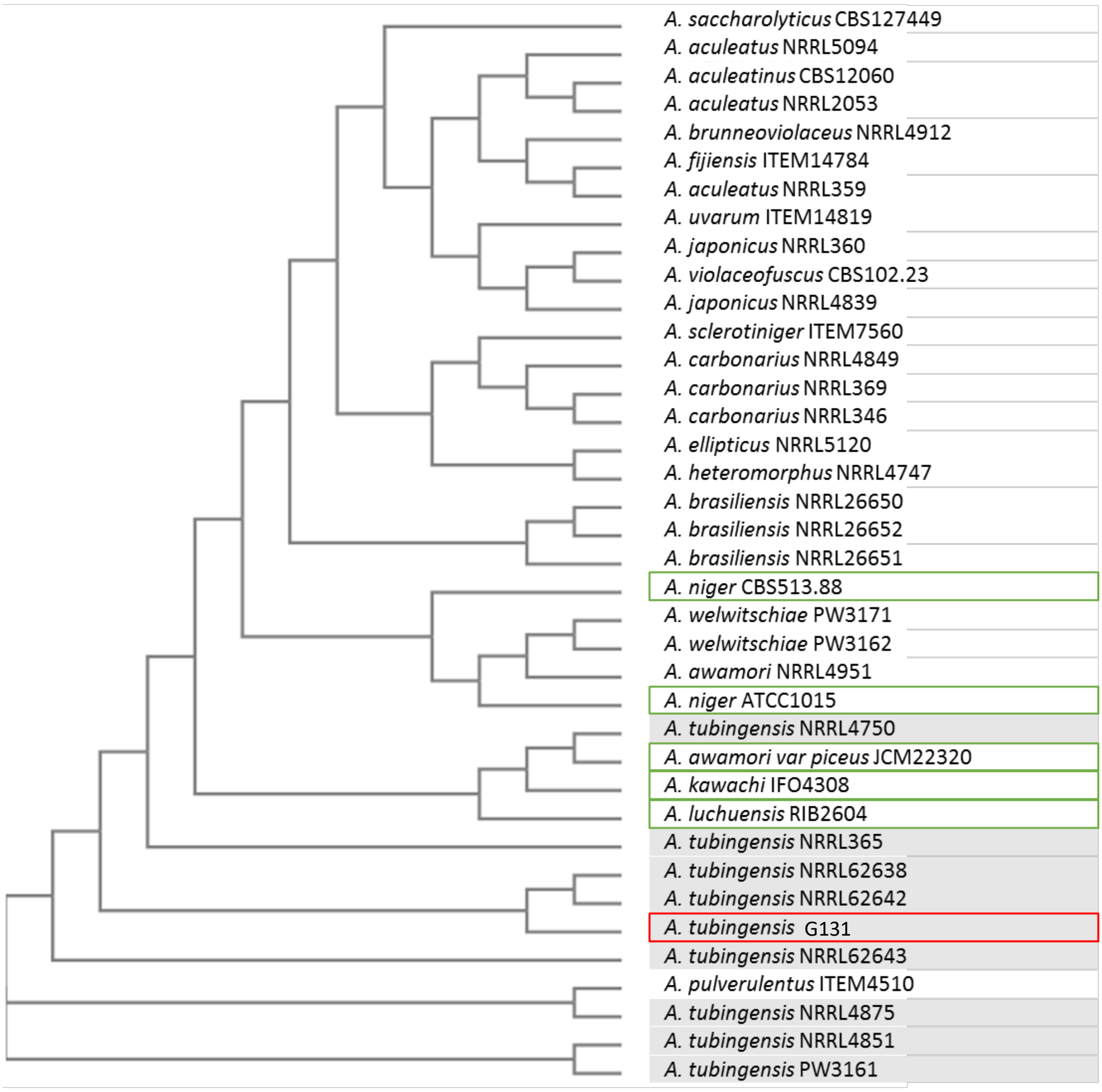

Supplementary Figure 3. Phylogenetic tree produced from *Cam1* partial gene sequence of 38 strains of black aspergilli. The evolutionary history was inferred using the Neighbour-Joining method computed with the Maximum Likelihood Evolutionary method. Grey background highlights *A. tubingensis* strains; Green frame highlights the genome used for the comparative analysis of this study; Red frame highlights the genome sequenced in this study.

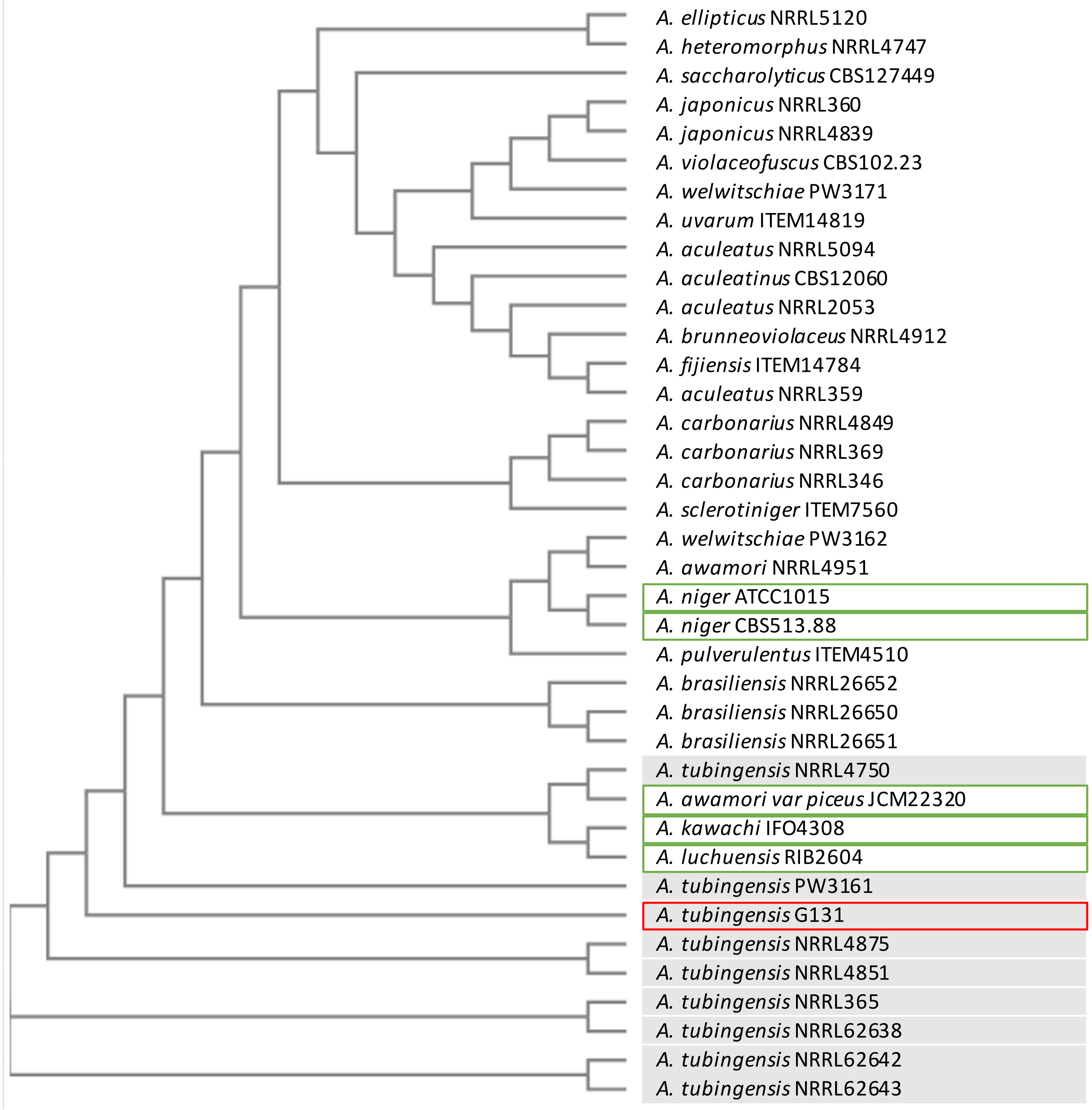

Supplementary Figure 4. Phylogenetic tree produced from *BenA* partial gene sequence of 38 strains of black aspergilli. The evolutionary history was inferred using the Neighbour-Joining method computed with the Maximum Likelihood Evolutionary method. Grey background highlights *A. tubingensis* strains; Green frame highlights the genome used for the comparative analysis of this study; Red frame highlights the genome sequenced in this study.

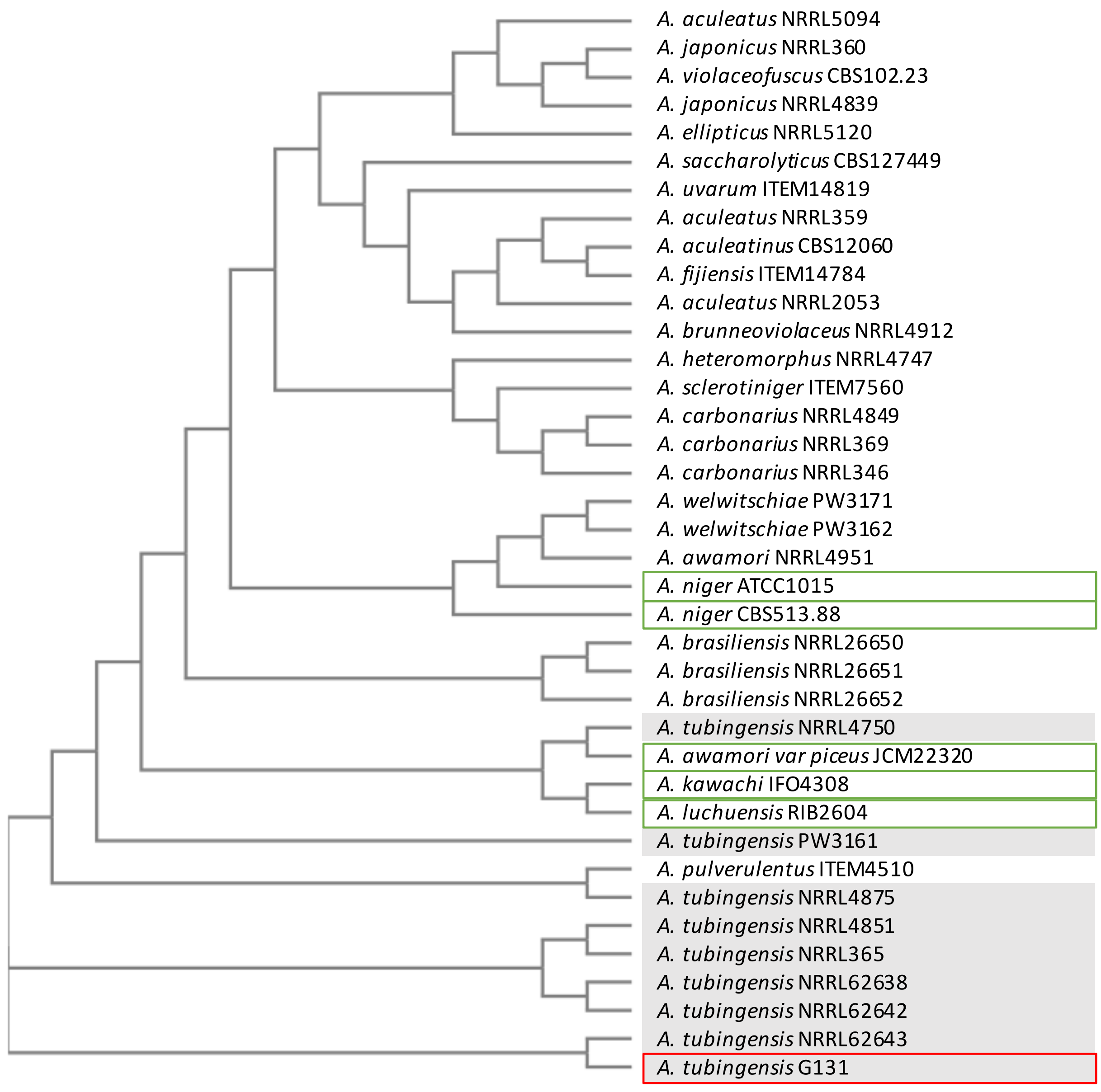

Supplementary Figure 5. Blast2GO statistics summary. **(A)** Graphical representation of the numbers of proteins sequences depending on their amino acid length. **(B)** Sequence similarity distribution according to NCBI tBLASTn. **(C)** Top-hit species distribution according to NCBI tBLASTn maximum similarity.

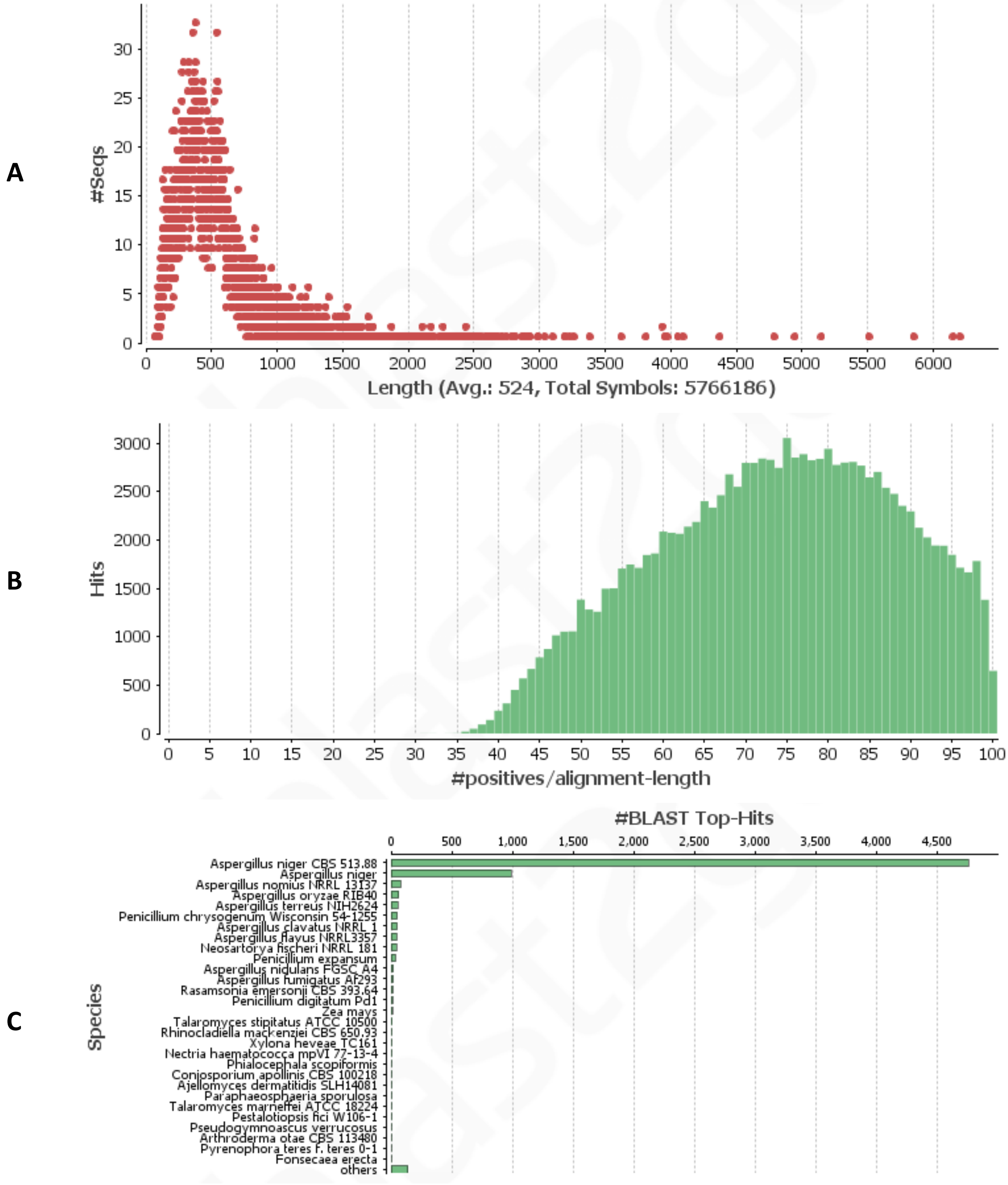

Supplementary Table 3. KOG analysis of *A. tubingensis* G131, *A. tubingensis* CBS 134.48, *A. kawachii* IFO 4308 and *A. niger* CBS 513.88

			A. tubingensis G131	*A. tubingensis* CBS134.48	*A. kawachii* IFO4308	*A. niger* CBS513.88
Intracellular Process	M	Cell wall/membrane/envelope biogenesis	140	133	134	138
	N	Cell motility	30	31	28	28
	O	Posttranslational modification, protein turnover, chaperones	259	257	255	258
	T	Signal transduction mechanisms	313	300	311	322
	U	Intracellular trafficking, secretion, and vesicular transport	22	24	23	21
	V	Defense mechanisms	100	104	90	97
	W	Extracellular structures	0	0	0	0
	Y	Nuclear structure	0	0	0	0
	Z	Cytoskeleton	5	6	6	6
Metabolism	C	Energy production and conversion	327	328	312	325
	D	Cell cycle control, cell division, chromosome partitioning	32	32	31	37
	E	Amino acid transport and metabolism	437	440	437	433
	F	Nucleotide transport and metabolism	113	114	117	118
	G	Carbohydrate transport and metabolism	776	785	764	759
	H	Coenzyme transport and metabolism	314	312	310	305
	I	Lipid transport and metabolism	484	485	473	486
	P	Inorganic ion transport and metabolism	195	198	190	191
	Q	Secondary metabolites biosynthesis, transport and catabolism	432	435	412	410
Information storage / processing	A	RNA processing and modification	2	2	2	0
	B	Chromatin structure and dynamics	5	5	5	5
	J	Translation, ribosomal structure and biogenesis	341	345	344	354
	K	Transcription	90	98	85	91
	L	Replication, recombination and repair	152	146	148	152
	X	Mobilome: prophages, transposons	6	4	13	14
Function poorly characterized	R	General function prediction only	531	549	528	532
	S	Function unknown	113	125	122	141

Supplementary Figure 6. Venn Diagram of secretome analysis from the four genomes obtained through SignalP and OrthoMCL analysis.

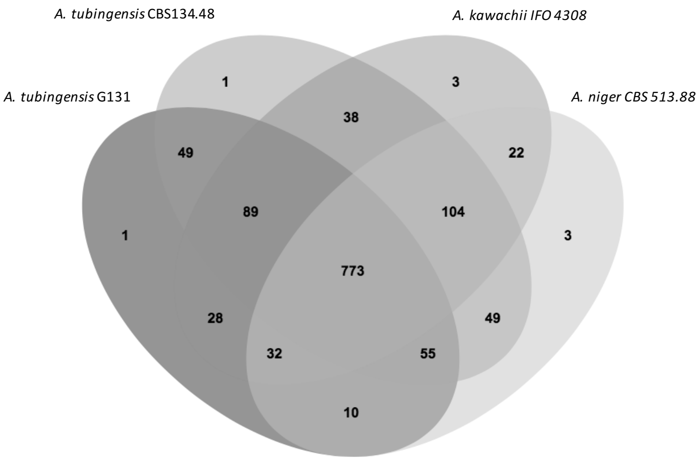

Summary

Species	Proteins	Clusters	Singletons
131	1107	1037	59
134	1391	1158	221
kaw	1204	1089	100
nig	1307	1048	247

Supplementary Table 4. SM Clusters coordinates of the scaffolds assembly

Cluster	Scaffold	Type	From	To	Size (Mb)	Software prediction
**Cluster 1**	1	PKS	912395	959260	46,8	AntiSMASH/SMURF
**Cluster 2**		Indole / DMAT	1882016	1903593	21,5	AntiSMASH
**Cluster 3**	2	NRPS	545874	591531	45,6	AntiSMASH/SMURF
**Cluster 4**		PKS	1797908	1839757	41,8	SMURF
**Cluster 5**	3	PKS	499150	541571	42,4	SMURF
**Cluster 6**		Terpene	546590	567902	21,3	AntiSMASH/SMURF
**Cluster 7**		PKS	1177242	1233449	56,2	SMURF
**Cluster 8**	4	NRPS / PKS	1	54029	54,0	AntiSMASH/SMURF
**Cluster 9**		PKS - Like	216512	229122	12,6	AntiSMASH/SMURF
**Cluster 10**		Terpene	943596	966557	22,9	AntiSMASH
**Cluster 11**		NRPS-Like	1142893	1185664	42,7	AntiSMASH/SMURF
**Cluster 12**		PKS	1320967	1350864	29,8	AntiSMASH/SMURF
**Cluster 13**	5	PKS	145883	183610	37,7	AntiSMASH/SMURF
**Cluster 14**		NRPS / PKS	247460	313730	66,2	AntiSMASH/SMURF
**Cluster 15**		NRPS-Like	321182	375703	54,5	AntiSMASH
**Cluster 16**		PKS	385775	427849	42,0	AntiSMASH/SMURF
**Cluster 17**		PKS	450165	502116	51,9	AntiSMASH/SMURF
**Cluster 18**		NRPS	855873	917813	61,9	AntiSMASH
**Cluster 19**		NRPS / PKS	959497	1030983	71,4	AntiSMASH/SMURF
**Cluster 20**		PKS	1129031	1175761	46,7	AntiSMASH/SMURF
**Cluster 21**		PKS	1182144	1264696	82,5	AntiSMASH/SMURF
**Cluster 22**	6	PKS / NRPS	52189	118782	66,5	AntiSMASH
**Cluster 23**	7	NRPS	77504	140216	62,7	AntiSMASH/SMURF
**Cluster 24**	8	NRPS	116290	157877	41,5	AntiSMASH/SMURF
**Cluster 25**		NRPS / PKS	669004	746754	77,7	AntiSMASH
**Cluster 26**	9	PKS	61364	102355	40,9	AntiSMASH/SMURF
**Cluster 27**		NRPS	256758	330233	73,4	AntiSMASH
**Cluster 28**	10	PKS-Like	147169	162478	15,3	AntiSMASH
**Cluster 29**		NRPS-Like	313518	363993	50,4	AntiSMASH/SMURF
**Cluster 30**	11	NRPS	134303	193931	59,6	AntiSMASH
**Cluster 31**		PKS	341757	401914	60,1	AntiSMASH/SMURF
**Cluster 32**	12	PKS	1	35618	35,6	AntiSMASH/SMURF
**Cluster 33**	13	PKS	503242	551263	48,0	AntiSMASH
**Cluster 34**	15	Terpene	33268	55292	22,0	AntiSMASH/SMURF
**Cluster 35**		NRPS-Like	55665	118723	63,0	AntiSMASH/SMURF
**Cluster 36**		PKS	456331	506832	50,5	AntiSMASH/SMURF
**Cluster 37**	17	Terpene	318637	340778	22,1	AntiSMASH/SMURF
**Cluster 38**		NRPS-Like	421674	495091	73,4	AntiSMASH/SMURF

**Cluster 39**	18	NRPS-Like	38728	93556	54,8	AntiSMASH/SMURF
**Cluster 40**	20	Other	1	29972	29,9	AntiSMASH/SMURF
**Cluster 41**		PKS	17198	62529	45,3	AntiSMASH
**Cluster 42**	22	NRPS	15447	62707	47,2	AntiSMASH/SMURF
**Cluster 43**		NRPS	253870	297885	44,0	SMURF
**Cluster 44**	24	PKS	134944	167775	32,8	AntiSMASH/SMURF
**Cluster 45**		PKS	169610	228959	59,3	AntiSMASH/SMURF
**Cluster 46**	25	PKS / NRPS	377354	438111	60,7	AntiSMASH/SMURF
**Cluster 47**		PKS	420050	477841	57,7	AntiSMASH/SMURF
**Cluster 48**	28	PKS	68141	116049	47,9	AntiSMASH
**Cluster 49**		PKS	262897	331308	68,4	AntiSMASH/SMURF
**Cluster 50**	30	PKS	92689	139212	46,5	AntiSMASH
**Cluster 51**	31	NRPS / PKS	84965	187441	102,4	AntiSMASH/SMURF
**Cluster 52**		PKS	271685	355531	83,8	AntiSMASH/SMURF
**Cluster 53**	32	Terpene	107326	129008	21,6	AntiSMASH/SMURF
**Cluster 54**		PKS	279629	324330	44,7	AntiSMASH/SMURF
**Cluster 55**	34	NRPS-Like	40243	94106	53,8	AntiSMASH/SMURF
**Cluster 56**		PKS / NRPS	329627	370978	41,3	AntiSMASH/SMURF
**Cluster 57**	35	PKS	87986	130141	42,1	AntiSMASH/SMURF
**Cluster 58**		NRPS	186883	264703	77,8	AntiSMASH/SMURF
**Cluster 59**		PKS	280407	327063	46,6	SMURF
**Cluster 60**	36	PKS / NRPS	1	35101	35,1	AntiSMASH/SMURF
**Cluster 61**		NRPS / PKS	214394	293555	79,1	AntiSMASH/SMURF
**Cluster 62**	37	Terpene	165840	186675	20,8	AntiSMASH/SMURF
**Cluster 63**		NRPS-Like	281517	338322	56,8	AntiSMASH/SMURF
**Cluster 64**	38	PKS	147939	218376	70,4	AntiSMASH/SMURF
**Cluster 65**	39	NRPS	117163	173941	56,7	AntiSMASH/SMURF
**Cluster 66**	40	PKS / NRPS	26203	89259	63,0	AntiSMASH/SMURF
**Cluster 67**		NRPS-Like	148178	189166	40,9	AntiSMASH/SMURF
**Cluster 68**	45	NRPS-Like	223303	257202	33,8	AntiSMASH/SMURF
**Cluster 69**	50	Indole / DMAT	114815	136265	21,4	AntiSMASH/SMURF
**Cluster 70**	54	NRPS-Like	130492	183294	52,8	AntiSMASH/SMURF
**Cluster 71**	55	Terpene	64407	84786	20,3	AntiSMASH/SMURF
**Cluster 72**	59	NRPS / PKS	1	43836	43,8	AntiSMASH/SMURF
**Cluster 73**	60	NRPS	23645	69365	45,7	AntiSMASH/SMURF
**Cluster 74**	61	NRPS	28510	104410	75,9	AntiSMASH/SMURF
**Cluster 75**	63	Terpene / PKS	65835	116629	50,7	SMURF
**Cluster 76**	68	NRPS	583	46109	45,5	AntiSMASH/SMURF
**Cluster 77**	71	NRPS	25287	79984	54,6	AntiSMASH
**Cluster 78**	75	NRPS-Like	43588	71160	27,5	AntiSMASH
**Cluster 79**	81	Terpene	36416	58580	22,1	AntiSMASH
**Cluster 80**	89	PKS	1	24173	24,1	AntiSMASH/SMURF

Supplementary Figure 7. Genes similarity between SM cluster 44 from *A. tubingensis* G131 with others known fungal biosynthetic cluster. (Results obtained with AntiSMASH software).

DH: dehydrogenase conserved domain; TR: transporter conserved domain; PKS: Polyketide synthase conserved domain; Kin: Kinase conserved domain

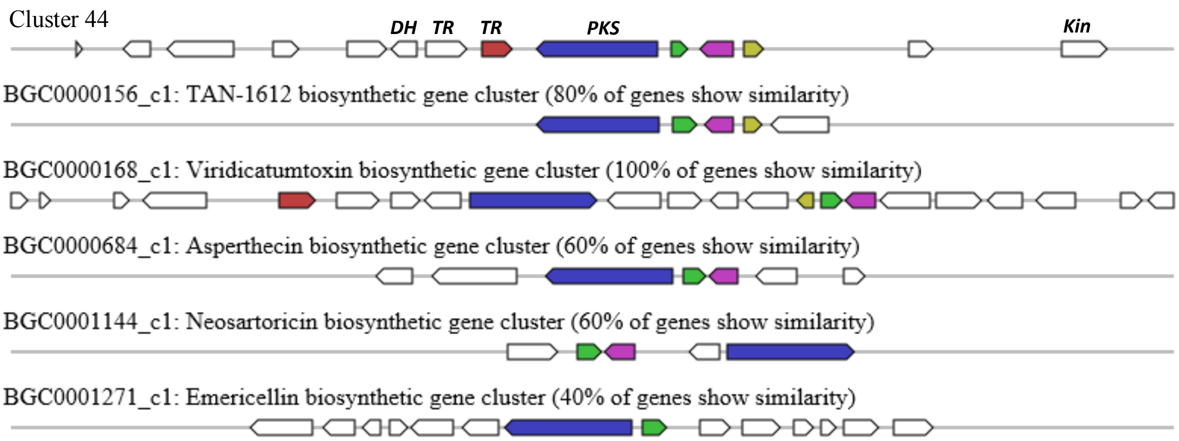

Supplementary Figure 8. AntiSMASH schematic representation of the conserved domains in SM clusters unique in *A. tubingensis* G131 and their similarities with other SM clusters identified in various fungi (AntiSMASH results).

450: Cytochrome P450 conserved domain; Glu: Glucosidase conserved domain; Hy: Hydratase conserved domain; Hyd: Hydrolase conserved domain; NRPS: Non Ribosomal Peptide Synthase conserved domain; OMet: o-methyltransferase conserved domain; PKS: Polyketide synthase conserved domain

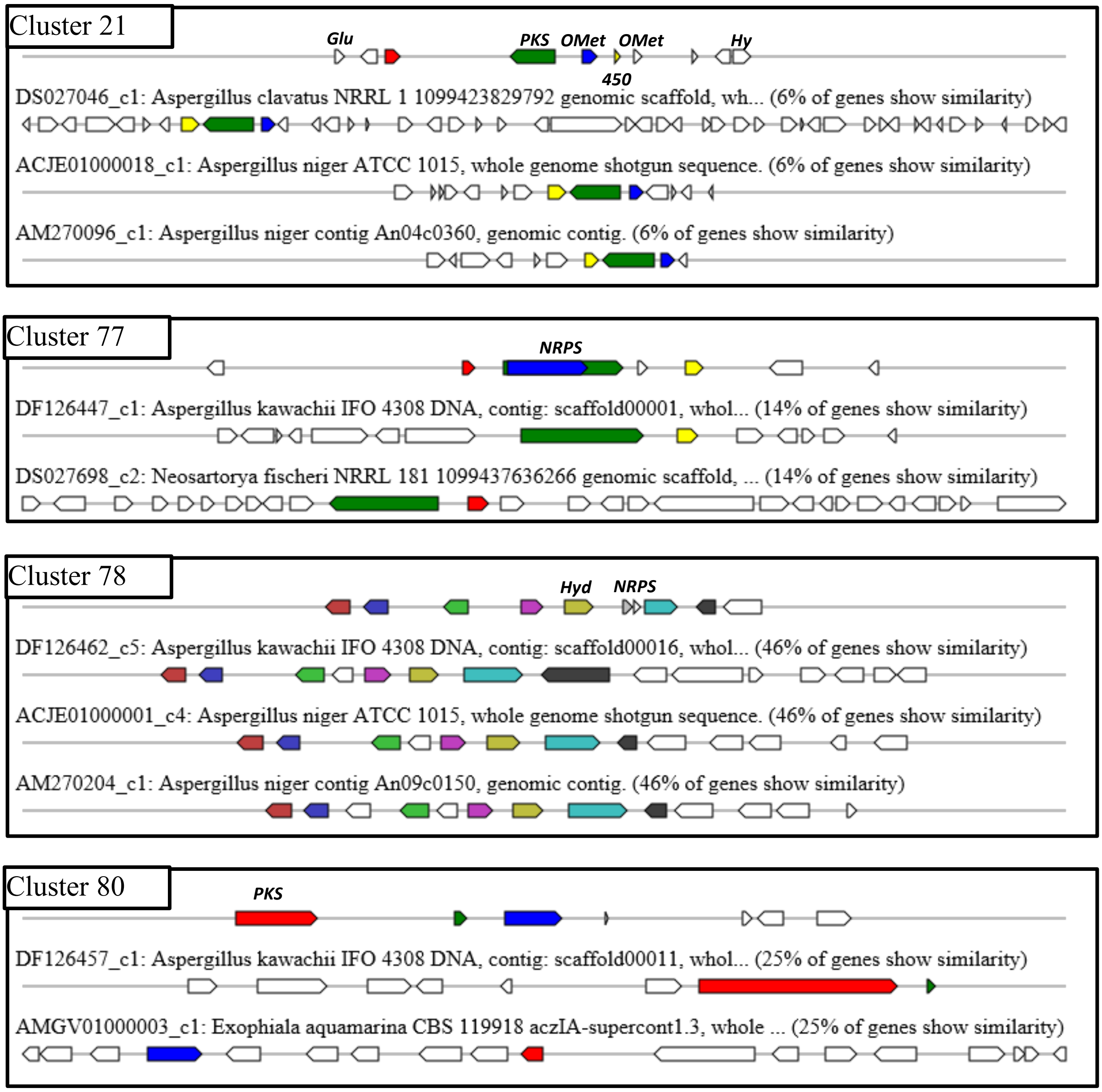

Supplementary Table 5. Blast results for Ochratoxin A, Fumonisins and asperazine Biosynthesis in *A. tubingensis*. Results were obtained on *A. tubingensis* G131 scaffolds assembly with blastall software. S1 is position of Scaffold 1. Only results with more than 5% coverage are shown here, under this n.d. is written for none detected. For TBlastN positivite matches (P.) is also indicated.

Name or function	Organism	NCBI accession	Blast Type	*A. tubingensis* top-hits results		
				Locus	Cov	Id
**OTA Biosynthesis**						
OTA putative cluster	*A. welwitschiae* ITEM 7468<br>*A. niger* ITEM 10355	KX267735<br>KX267737<br>KX267736	BlastN	S8:<br>59674 - 70316	57%	54%
OTA PKS	*P. verrucosum*	DQ789993	BlastN	n.d	n.d.	n.d.
Halogenase	*A. carbonarius* ITEM 5010	KU960948	BlastN	n.d	n.d.	n.d.
OTA NRPS	*P. nordicum*	AY557343	BlastN	n.d	n.d.	n.d.
Hog1	*P. nordicum*	KC618447	BlastN	S61:<br>60188 – 60480	18%	84%
Hog1	*P. verrucosum*	KC618446	BlastN	S61:<br>60188 – 60480	18%	84%
NRPS-like	*A. flavus* NRRL 3357	XM002383511	BlastN	S19:<br>72304 - 73291	26%	22.5%
**Fumonisins Biosynthesis**						
Fumonisin cluster	*A. niger* ITEM 10355	KJ934797	BlastN	S2:<br>1806873 - 1835919	53%	44%
Fum1	*A. niger* ITEM 4501	LK931893	BlastN	S2:<br>1821910 - 1822468	83%	28%
Fum3	*A. niger* ITEM 12918	LK931958	BlastN	n.d	n.d.	n.d.
Fum7	*A. niger* ITEM 4501	LK931878	BlastN	n.d	n.d.	n.d.
Fum8	*A. niger* ITEM 4501	FN662673	BlastN	n.d	n.d.	n.d.
Fum10	*A. niger* ITEM 4501	LK931969	BlastN	n.d	n.d.	n.d.
Fum13	*A. niger* ITEM 4501	LK931983	BlastN	n.d	n.d.	n.d.
Fum14	*A. niger* ITEM 4501	LK931921	BlastN	n.d	n.d.	n.d.
Fum15	*A. niger* ITEM 12918	LK932002	BlastN	S2:<br>1825981 - 185919	9%	87%
Fum21	*A. niger* ITEM 4501	LK931936	BlastN	n.d	n.d.	n.d.
**Asperazine Biosynthesis**						
Fumitremorgin cluster	*A. fumigatus*	AB436628.1	BlastN	S25:<br>402354 - 438111	44%	89%
DptA	*A. flavus* NRRL 3357	B8NR69	TBlastN	S25:<br>398244 - 407876	92%	28%<br>P. 46%
DptB	*A. flavus* NRRL 3357	B8NR70	TBlastN	S25:<br>380741 - 381117	97%	28%<br>P. 43%
DptC	*A. flavus* NRRL 3357	B8NR71	TBlastN	nd.	nd.	nd.

Supplementary Figure 9. Schematic representation of Cluster 4 presenting small homology with fumonisin cluster.

Arrows indicate putative genes in the cluster and its direction indicates forward and reverse strand. Blue: gene coding for backbone enzyme, Red: gene coding for enzymes with known function, Green: gene coding for transcription factor, Yellow: gene coding for transporter, Grey: gene coding for hypothetical protein. Bleu frame indicate region showing homology with fumonisin cluster of *A. niger* ITEM 10355.

Below each biosynthetic gene, the putative function is indicated. OR: oxydoreductase; Tr: transferase; MFS: MFS transporter; PKS: Polyketide synthase; Dhy: Dehydrogenanse

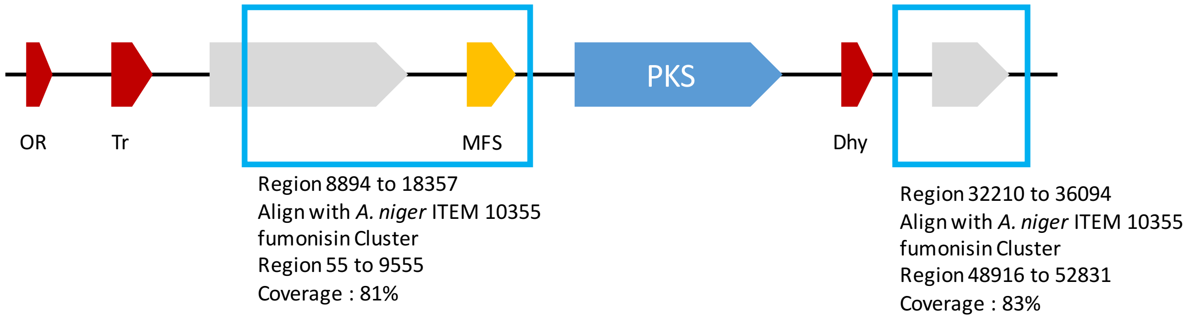

Supplementary Figure 10. Repartition of conserved domains in predictive enzymes coding genes of the asperazine obtain with CD search on the NCBI

- Putative NRPS conserved domains

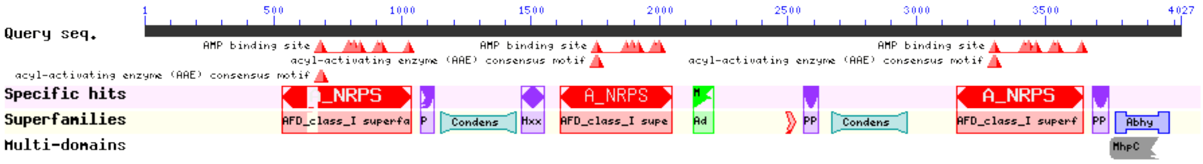

- Putative PKS conserved domains

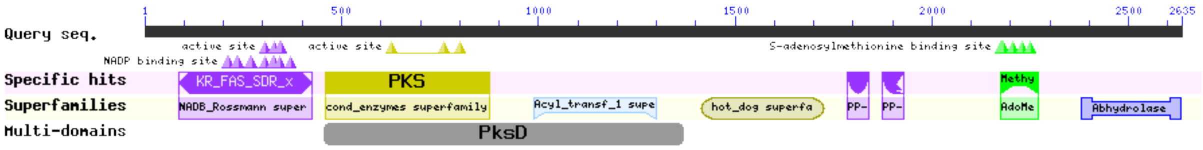

- Putative methyltransferase conserved domains

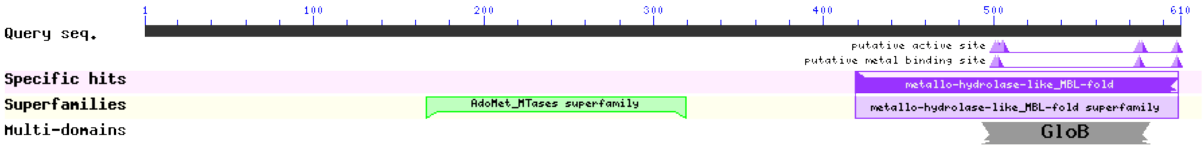

- Putative Cytochrome P450 conserved domains (Figure 4B – 450-1)

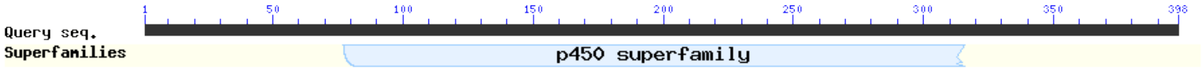

- Putative Cytochrome P450 conserved domains (Figure 4B – 450-2)

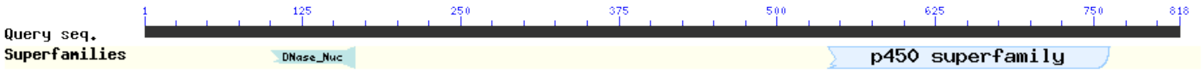

Supplementary Figure 11. Conserved domain (CD-Search) and Distance tree results obtained from NCBI BlastP analysis of predicted PKS of *A. tubingensis* G131 putatively involved in NGPs synthesis (Neighbour-Joining, maximum distance >0.5; grisham mode) **A.** Comparison between conserved domains of *A. tubingensis* G131 SM Cluster 16 prediction and other known fungal genome assemblies – representation obtained with AntiSMASH software. **B.** Putative PKS from Cluster 54.

A. Cluster 16 PKS

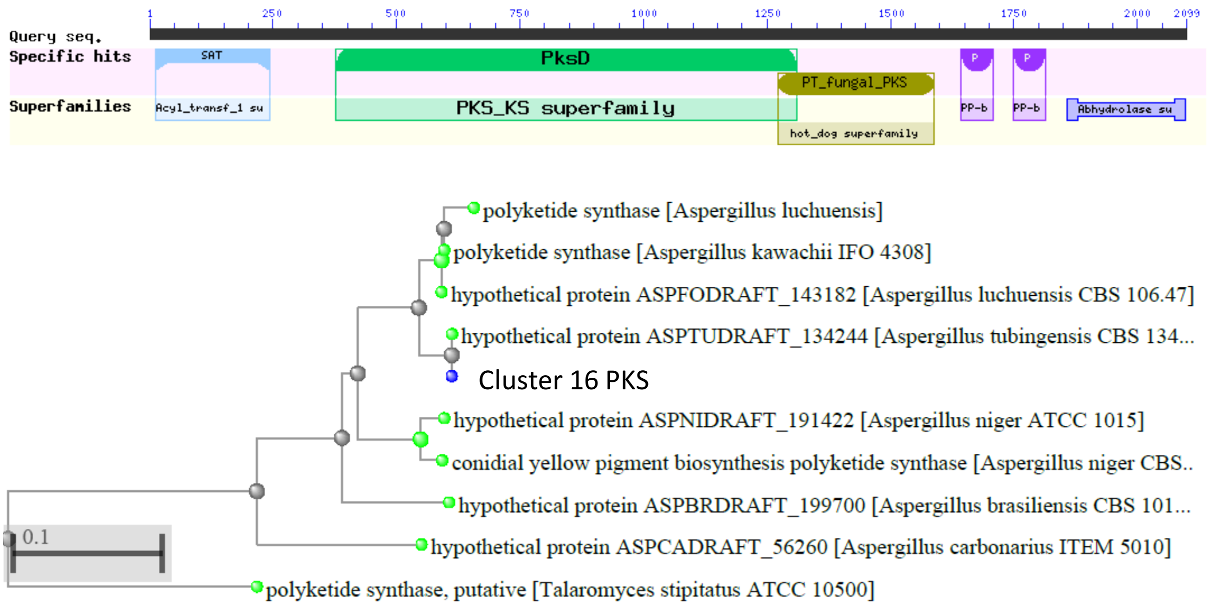

B. Cluster 54 PKS

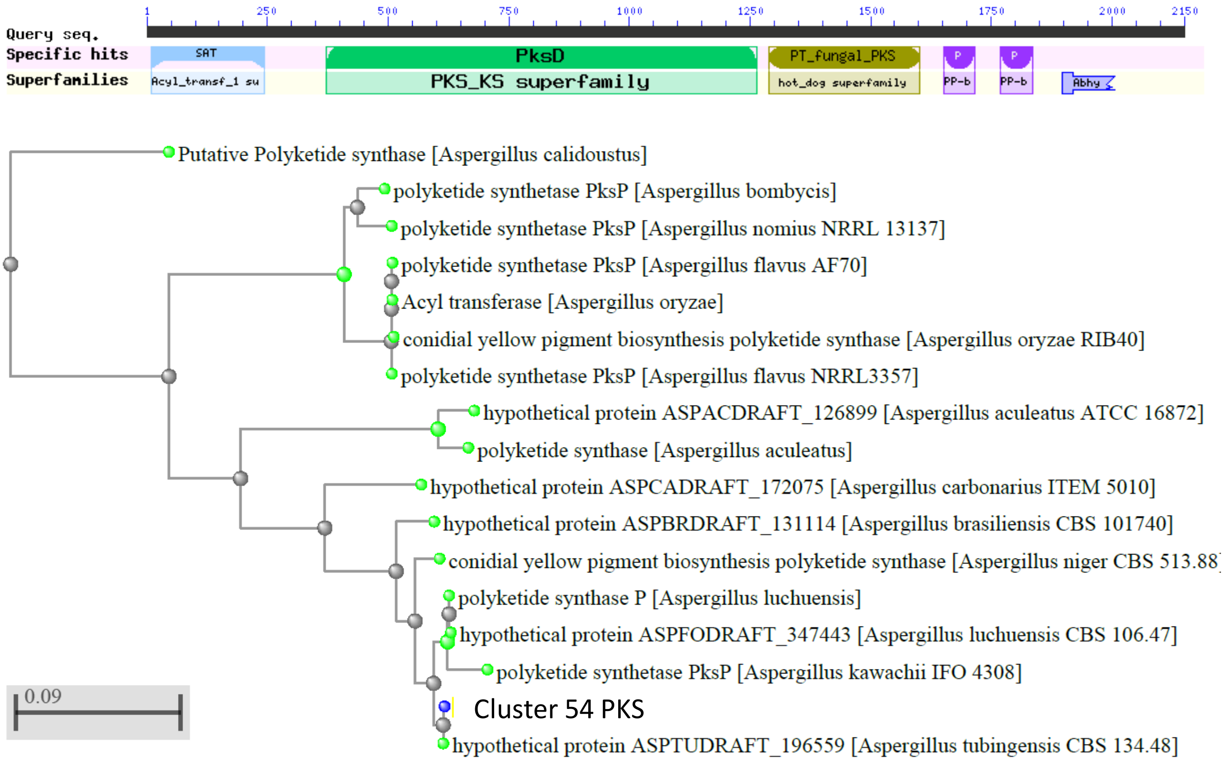

Supplementary Figure 12. Clustal Omega Alignment of predicted PKS from SM Clusters 54 and 16 of *A. tubingensis* G131.

```
Cluster 16 PKS      MSKMETVAEVFIFGDQTVAFESTLHSLLHVKDNAVLTDFFDRVGYQLRRYVGNLPLHQQE
Cluster 54 PKS      ---MEGPSRVYLFGDQTSDIEAGLRRLLQAKNSTIVQSFFQQCFHAIRQEIAKLPPSQRK
                       **  :.*::*****  :*: *: **:.*:.::: .**::  : :*: :.:**  *::

Cluster 16 PKS      WFPYFTTLIDLLAQHENCHAAPALKLPLLCATELGQFIRHYGQAGRTYPAPESTFLIGAC
Cluster 54 PKS      LFPRFTSIVDLLSRSRESGPSPVLESALTCIYQLGCFIHFYGDLGHDYPTPSNSHLVGLC
                      ** **:::***:: .:.  :*.*:  * *  :** **:.**: *: **:*..:.*:* *

Cluster 16 PKS      TGAFAAAAVATSQTLAELVPAAVEAVTVAFRTALRSLILRNDLELNIDGSQKSWSAIV-G
Cluster 54 PKS      TGVLSCTAVSCAKNVGELIPAAVEAIVIALRLGICVFRVRE-LVASADSESTCWSALVSG
                    **.::.:**: ::.:.**:******:.:*:* .:  : :*: *  . *.....***:* *

Cluster 16 PKS      VCEPEAASAIRCFNVEKGLPPIARLYLSSVSRTNVSISGPPRLLGQFLLSCA---WKHSL
Cluster 54 PKS      ISEAEASRLIDEYSSKKATPPSSKPYISAVSSNGVTVSAPPTILDEFVETCISKNYKPVK
                    :.* **:  *  :. :*. ** :: *:*:** ..*::*.** :*.:*: :*    :*

Cluster 16 PKS      LPIELPYHASHLFRKDDVEIIVGDFHDFS-LLKYTQRTKVICAVSGEVVVASDLRSLLHR
Cluster 54 PKS      APIHGPYHAPHLYDDKDIERILHQSSALEELTGWSPAIPVISSNTGKPIKATSIKDLFKV
                     **. **** **: ..*:* *: :   :. *  ::    **.: :*: : *:.::.*::

Cluster 16 PKS      AVQEALCEPLQWNCIAASLSRELKQCQLTECVINQVSTRSGSLISSALSQEAFLGVKIVQ
Cluster 54 PKS      ALEEILLRRLCWDKVTESCTSVCKTGKNHSCKLFPISSSATQSLFT-VLKKAGVSISLET
                    *::* * . * *: :: * :   *  :  .* :  :*: : . : : : ::* :.:.:

Cluster 16 PKS      SIGDVAVSPALSSPTGKFSQSSIAIIGFSGRFPESDSNEEFWEMLLAGRDVHRTIPEDRF
Cluster 54 PKS      GVGEIAINPETRNLTGKAENSKIAIIGMSGRFPESDGTEAFWDLLYKGLDVHRKVPADRW
                    .:*::*:.*    . *** .:*.*****:********..* **::*  * ****.:* **:

Cluster 16 PKS      DWKAHYDPTGKSKNTSRVKYGCFIKEPGVFDARFFNMSPREAENADPAQRLAITTAYEAM
Cluster 54 PKS      DVDAHVDMTGSKRNTSKVSYGCWINEPGMFDPRFFNMSPREALQADPAQRLALLTAYEAL
                    * .** * **..:***:*.***:*:***:** ********** :********: *****:

Cluster 16 PKS      EMAGLVPNATPSTQGDRIGVFYGVTSDDWREVNSGQNVDTYFIPGGNRAFIPGRISYFFR
Cluster 54 PKS      EMAGFIPDSSPSTQRDRVGIFYGMTSDDYREINSGQDIDTYFIPGGNRAFTPGRINYYFK
                    ****::*:::**** **:*:***:****:**:****::************ ****.*:*:

Cluster 16 PKS      FCGPSLSIDTACSSSCAAIQTACAYLWRGECDTALAGGVNVLTNPDNFCGLDRGHFLTAK
Cluster 54 PKS      FSGPSVSVDTACSSSLAAIHMACNSIWRNDCDAAITGGVNILTNPDNHAGLDRGHFLSTT
                    *.***:*:******* ***: **  :**.:**:*::****:******..********::.

Cluster 16 PKS      GNCNAFDDEADGYCRSDAVGTVILKRLEDALEDSDPIFGVIRGAYTNHCGRTDSITRPFE
Cluster 54 PKS      GNCNTFDDGADGYCRADGVGSIVLKRLEDAEADNDPILAVINGAYTNHSAEAVSITRPHV
                    ****:*** ******:*.**:::*******  *.***:.**.******...: *****.

Cluster 16 PKS      GDQATVFNRIMRYAGVDPQDVGYVEMHGTGTQAGDATEMKSVLSVFAPNMRRQ--NPLYL
Cluster 54 PKS      GAQAFIFNKLLNDANIDPKDVSYVEMHGTGTQAGDAVEMQSVLDVFAPDYRRGPGQSLHI
                    * ** :**:::. *.:**:**.**************.**:***.****: **   : *::

Cluster 16 PKS      GTAKANMGHAESASGVSSLIKVLLMMRNDMIPPHCGIKTRINHTYPVDLEQRNVHIPFKP
Cluster 54 PKS      GSAKANIGHGESASGVTALVKVLLMMRENMIPPHCGIKTKINSNFPTDLAKRNVHIAFQP
                    *:****:**.******::*:*******::*********:** .:*.** :***** *:*

Cluster 16 PKS      TPWLREQMPQFKRTVFLNNFSAAGGNTAMLIEDAPIRERKLAADPRSKHVVTVTAKTIKA
Cluster 54 PKS      TPWNRPAS--GKRRTFVNNFSAAGGNTALLVEDAPIPERQ-GQDPRSFHLVSVSARSQSA
                    *** *      ** .*:**********:*:***** **: . **** *:*:*:*:: .*

Cluster 16 PKS      LKGNISSLIAYLEQHP-------GVSLSSLSYTTTARRIHHSYRVIVSGSDTGEFLHRMQ
Cluster 54 PKS      LKNNVEALVKFIDAQGKSFGVKETEFLPNLAYTTTARRIHHPFRVTAVGANLQSLRDSL-
                    **.*:.:*: ::: :           * .*:********** :** . *::  .: . :

Cluster 16 PKS      EILPGLESHRLVPPPGKLPNIVMTFTGQGTLYVGMGKQFYDTVPSFRADVMRFNEIASQM
Cluster 54 PKS      --HGALNRETYTPVPSTAPGIGFVFTGQGAQYTGMGKELYRSCFQFRTTIEHFDCIARSQ
                         .*: .  .* *.. *.* :.*****: *.****::* :  .**: : :*: ** .

Cluster 16 PKS      GFPSFLPLIDGTMINHQEASPTVAHLALVCIQMALYNHWMSLGVIPSANIGHSLGEYPAL
Cluster 54 PKS      GLPSILPLVDGSVP-VEDLSPVVVQVGTTCVQMALVNYWTALGVKPAFIIGHSLGDYAAL
                    *:**:***:**::   :: **.*.::. .*:**** *:*  *** *:  ******:* **

Cluster 16 PKS      YASGVLTASSVIYLVGARATLLAEETTPRTHAMLAVKGN---------------------
Cluster 54 PKS      NTAGVLSTSDTIYLCGRRAQLLTKECKIGTHSMLAIKASLAEVKQFLRDELHEVSCINAP
                     ::***::*..*** * ** **::* .  **:***:*..
```

```
Cluster 16 PKS      ---VVSGSIKELMQLKDWLMSRGIESVQLDIPYAFHSAQVDPILDDFMAIAAGVHYYVPS
Cluster 54 PKS      AETVVSGLVADIDELAQRCSTEGLKSTKLRVPYAFHSSQVDPILDSFEDIAQGVTFHKPT
                       **** : :: :* :   :.*::*.:* :******:*******.*  ** ** :: *:

Cluster 16 PKS      IPYISPLLSRVIPNGEPNVFGACYLKNACREPVNFQGAVEAAEKTGLVNEKTIWVDIGSH
Cluster 54 PKS      TPFVSALFGEVITDANWECLGPKYLRDHCRKTVNFLGGVEATRHAKLTNDKTLWVEIGSH
                     *::* *:..** :.: : :*  **:: **: *** *.***:.:: *.*:**:**:****

Cluster 16 PKS      PACSGMVKGILGAKSVAIASLRKGTDSWSVLSAGLEVLYSHGIDIKWVEYHRGIVGAREV
Cluster 54 PKS      TICSGMIKATLGPQVTTVASLRREEDTWKVLSNSLASLHLAGIDINWKQYHQDFSSSLQV
                      ****:*. ** : .::****:  *:*.*** .*  *:  ****:* :**:.: .: :*

Cluster 16 PKS      LQLPRYDWDLKNYWIQYRNNFCLLKGEGLVPAGQQAVSPHMEFPRIPQYLSPSVQRVLEQ
Cluster 54 PKS      LRLPGYKWDLKNYWIPYTNNFCLSKGAPVAAVA---AGPQ------HEFLTTAAQKVIET
                    *:** *.******** * ***** **  :. ..   ..*:       ::*: :.*:*:*

Cluster 16 PKS      NDGPETSTLLAESDIHDARLAPILAGHVVNGAMLCPSSLYADIGITISKHMLEAIGKYTD
Cluster 54 PKS      RGDGTTATVVIENDIADPDLNRVIQGHKVNGAALCPSSLYADISQTLAEYLIKKYKPEYD
                     ...  *:*:: *.** *  *  :: ** **** **********. *:::::::      *

Cluster 16 PKS      TVGLDVADMQVQNPLISRPDTESQVFHVSASADWRINGISFRLYSVNGNGKKTVEHAAYV
Cluster 54 PKS      GLGLDVCEVTVPRPLIAKG--GQQLFRVSATADWAEKKTTLQIYSVTAEGKKTADHATCT
                     :****.:: * .***::    .*:*:***:***  :  ::::***..:****.:**: .

Cluster 16 PKS      VHITEQVQTWLTEWKRHAHLVRSRIASLHRSAEEGDAHKLKRRLAYQLFATLVQYETSYQ
Cluster 54 PKS      VRLFDC-AAAEAEWKRVSYLVKRSIDRLHDIAEDGDAHRLGRGMVYKLFAALVDYDENFK
                    *:: :   :  :**** ::**:  *  **  **:****:* * :.*:***:**:*: .::

Cluster 16 PKS      GMQEVVLDCDNHEATAKVSFQTDENGFVFNPCWIDSLGHIAGFIMNATDATPSKKQVFIN
Cluster 54 PKS      NIREVILDSEQHEATARVKFQASQGKFHRNPFWIDSFGHLSGFIMNASDATDSKNQVFVN
                    .::**:**.::*****:*.**:.:. *  ** ****:**::******:*** **:***:*

Cluster 16 PKS      HGWERMRCAVRFDKGKQYQVYNRMQLETGTTYVGDTYIFEGETVVAVYQGIRFQGVARRL
Cluster 54 PKS      HGWDSMRCLKKFSPDVTYRTYVRMQPWKDSIWAGDVYVFDGEDIVAVYGAVKFQGLSRKI
                    ***: ***  :*. .  *:.* ***  ..: :.**.*:*:** :**** .::***::*::

Cluster 16 PKS      LDRLLPPKMQSRLADRNQEVQPTTAPVNNQPVCRTAGRPSVPIVPAPNPKEPVGSSGGIA
Cluster 54 PKS      LDTVLPPVGASKAPARPAASAQKAAP-----AAPSKSRASA---PAPAKPAAKPSAPSLA
                    ** :***   *:   *      .:**     .. : .* *.   ***       *: .:*

Cluster 16 PKS      ARVMAIIAKEAGVHPSDLGANEEFANYGIDSLLSLTICGRIQEELDVDVPASLFVDYPTP
Cluster 54 PKS      KRALTILAEEVGLSESEITDDLVFADYGVDSLLSLTVTGRYREELDIDLESSVFIDQPTV
                     *.::*:*:*.*:  *::  :  **:**:*******: ** :****:*: :*:*:* **

Cluster 16 PKS      KDLIGFFGVEDDSSQL---------TSSLEGSTEDDTSSY-ETVATS-----EHESDGSV
Cluster 54 PKS      KDFKQFLAPMSQGEVSDGSTSDPESSSSFNGGSSTDESSAGSPVSSPPNEKVTQVEQHAT
                    **:  *:.  .:..           :**::*.:. * **  . *::       : .: :.

Cluster 16 PKS      LEVLRTTIAQEAGVSTDELTPTTAFTDIGVDSLLALTIVSTISETYDITLPSNILMEKEN
Cluster 54 PKS      IKEIRAILADEIGVSEEELKDDENLGEMGMDSLLSLTVLGRIRETLDLDLPGEFFIENQT
                    :: :*: :*:* *** :**.    : ::*:****:**::. * ** *: **.::::*::.

Cluster 16 PKS      LEEVGKALGLEVNPKTSPQHLVLPKPAFHP-----------------SVGPQATSILLWG
Cluster 54 PKS      LNDVEDALGLKPKPAPAPAPAPAPVPAPVSAPILKEPVPNANSTIMTRASPHPRSTSILLQG
                    *::* .****: :*  :*    :* *.  *                 *  *::***** *

Cluster 16 PKS      KPKTARKILFLFPDGSGSATSYSALPKLGNDTAAYGLNCPWMKTPQQMTVSLEELTAKYL
Cluster 54 PKS      NPKTATKTLFLFPDGSGSATSYATIPGVSPDVCVYGLNCPYMKTPEKLKYPLAEMTFPYL
                    :**** * **************:::* :. *...******:****:::.  * *:*  **

Cluster 16 PKS      LEVRRRQPNGPYYLGGWSAGGICAYEAARQLAQD-GQTTAKLILIDSPNPVGMENPPKRM
Cluster 54 PKS      AEIRRRQPKGPYNFGGWSAGGICAYDAARYLILEENEQVDRLLLLDSPFPIGLEKLPTRL
                     *:*****:*** :***********:*** *  : .: . :*:*:*** *:*:*: *.*:

Cluster 16 PKS      YDFFQSIGLFGTSGKPPPGWLIPHFNAFIRLLDAYRIQPLGAS--------IETHIVYAR
Cluster 54 PKS      YGFINSMGLFGEGNKAPPAWLLPHFLAFIDSLDTYRAVPLPFDDPKWAKKMPKTFMVWAK
                    *.*::*:**** ..* **.**:*** ***  **:**  **  .         :*.:*:*:

Cluster 16 PKS      DGICKDASSPRPERRPD---DPREMVWLIENRVDFSGDGWASLLGRENLH-IEVLSEVNH
Cluster 54 PKS      DGICSKPDDPWPEPDPDGKPDTREMVWLLKNRTDMGPNKWDTLVGPQNVGGITVIEGANH
                    ****.. ..* **  **   * ******::**.*:. : * :*:* :*:  * *:. .**

Cluster 16 PKS      FSMMDPGEHMEAFEGFLCRALL-
Cluster 54 PKS      FTMT-LGPKAKELGSFIGNAMAN
                    *:*   * : : : .*: .*:
```

Supplementary Figure 13. A. Genes similarity between Cluster 16 from *A. tubingensis* G131 with other known fungal genomes assembly (results obtained with AntiSMASH software). **B.** Distance tree results obtained from NCBI BlastN analysis of Cluster 16 of *A. tubingensis* G131 putatively involved in NGPs synthesis (Neighbour-Joining, maximum distance >0.5)

PKS: Polyketide synthase conserved domain; OMet: o-methyltransferase conserved domain; Transf: transferase conserved domain

A.

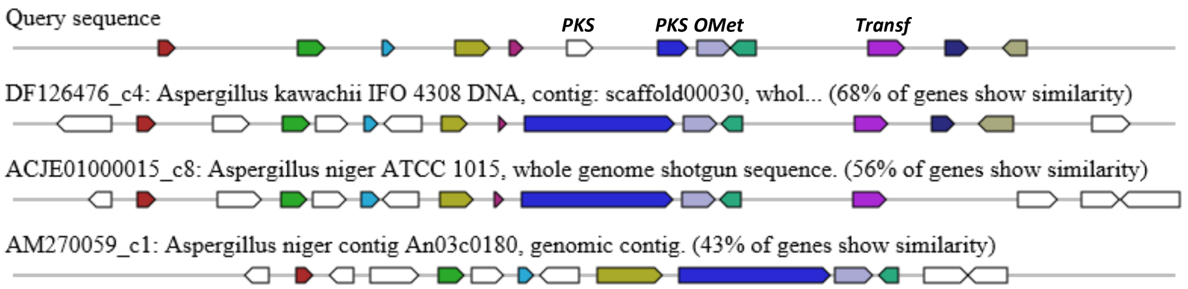

B.

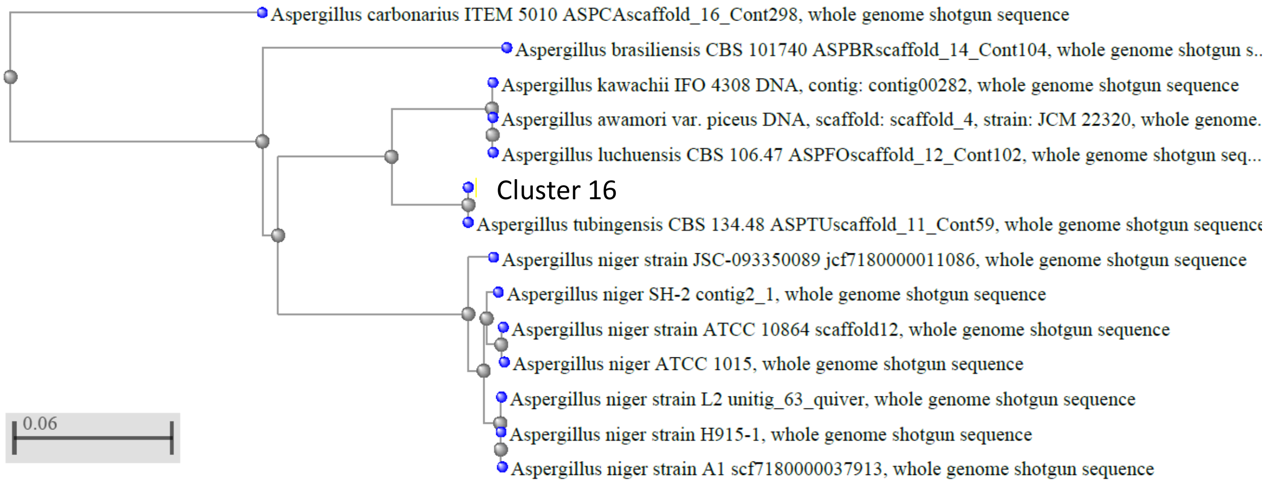

Supplement: Supplementary file 1 — Table S1. BUSCO analysis of A. tubingensis G131 scaffolds assembly. Figure S1. Dot Plot analysis between A. tubingensis G131 and A. tubingensis CBS 148.33 to order the scaffold assembly. Table S2. Accession number (NCBI) of sequences used for phylogenetic analysis. Figure S2. Phylogenetic tree produced from Rpb2 partial gene sequence of 38 strains of black aspergilli. Figure S3. Phylogenetic tree produced from Cam1 partial gene sequence of 38 strains of black aspergilli. Figure S4. Phylogenetic tree produced from BenA partial gene sequence of 38 strains of black aspergilli. Figure S5. Blast2GO statistics summary. Table S3. KOG analysis of A. tubingensis G131, A. tubingensis CBS 134.48, A. kawachii IFO 4308 and A. niger CBS 513.88. Figure S6. Venn Diagram of secretome analysis obtained through SignalP and OrthoMCL analysis. Table S4. SM Clusters coordinates of the scaffolds assembly. Figure S7. Genes similarity between SM cluster 44 from A. tubingensis G131 and others known fungal biosynthetic cluster. (Results obtained with AntiSMASH software). Figure S8. AntiSMASH schematic representation of the conserved domains in SM clusters unique in A. tubingensis G131 and their similarities with other SM clusters identified in various fungi (AntiSMASH results). Table S5. Blast results for Ochratoxin A, Fumonisins and asperazine biosynthesis in A. tubingensis. Figure S9. Schematic representation of Cluster 4 presenting small homology with fumonisin cluster. Figure S10. Repartition of conserved domains in predictive enzymes coding genes of the asperazine cluster obtained with CD search. Figure S11. Conserved domain (CD-Search) and Distance tree results obtained from NCBI BlastP analysis of predicted PKS of A. tubingensis G131 putatively involved in NGPs synthesis. Figure S12. Clustal Omega Alignment of predicted PKS from SM Clusters 54 and 16 of A. tubingensis G131. Figure S13. Genes similarity between Cluster 16 from A. tubingensis G131 with other known fungal genomes assembly [file 12864_2018_4574_MOESM1_ESM.pdf]
